# Drought imprints on crops can reduce yield loss: Nature's insights for food security

**DOI:** 10.1002/fes3.332

**Published:** 2021-09-30

**Authors:** Peng Fu, Deepak Jaiswal, Justin M. McGrath, Shaowen Wang, Stephen P. Long, Carl J. Bernacchi

**Affiliations:** ^1^ Carl R. Woese Institute for Genomic Biology University of Illinois at Urbana‐Champaign Urbana Illinois USA; ^2^ Departments of Plant Biology and Crop Sciences University of Illinois at Urbana‐Champaign Urbana Illinois USA; ^3^ USDA‐ARS Global Change and Photosynthesis Research Unit University of Illinois at Urbana‐Champaign Urbana Illinois USA; ^4^ Department of Geography and Geographic Information Science University of Illinois at Urbana‐Champaign Urbana Illinois USA; ^5^ Lancaster Environment Centre Lancaster University Lancaster UK

**Keywords:** climate change, crop phenology, drought priming, food security, temperature stress, US corn belt

## Abstract

The Midwestern “Corn‐Belt” in the United States is the most productive agricultural region on the planet despite being predominantly rainfed. In this region, global climate change is driving precipitation patterns toward wetter springs and drier mid‐ to late‐summers, a trend that is likely to intensify in the future. The lack of precipitation can lead to crop water limitations that ultimately impact growth and yields. Young plants exposed to water stress will often invest more resources into their root systems, possibly priming the crop for any subsequent mid‐ or late‐season drought. The trend toward wetter springs, however, suggests that opportunities for crop priming may lessen in the future. Here, we test the hypothesis that early season dry conditions lead to drought priming in field‐grown crops and this response will protect crops against growth and yield losses from late‐season droughts. This hypothesis was tested for the two major Midwestern crop, maize and soybean, using high‐resolution daily weather data, satellite‐derived phenological metrics, field yield data, and ecosystem‐scale model (Agricultural Production System Simulator) simulations. The results from this study showed that priming mitigated yield losses from a late season drought of up to 4.0% and 7.0% for maize and soybean compared with unprimed crops experiencing a late season drought. These results suggest that if the trend toward wet springs with drier summers continues, the relative impact of droughts on crop productivity is likely to worsen. Alternatively, identifying opportunities to breed or genetically modify pre‐primed crop species may provide improved resilience to future climate change.

## Graphical abstract

The United States Midwest is moving towards a climate pattern with wetter springs and drier mid‐ to late‐summers. This change in precipitation pattern, however, suggests that opportunities for crop priming may lessen in the future. Here, we quantify drought priming effects on maize and soybean and analyze what plant traits may associate with the observed priming effects in the field scale.

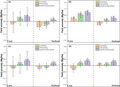

## 1 INTRODUCTION

Between 1960 and 2018, the United States, on average, accounted for 35% of global maize production and almost 50% of soybean production (FAO, 2019) with ca. 90% and 75% of maize and soybean, respectively, produced in the Midwest where irrigation is scarce (McGrath et al., 2015; USDA, 2017). These maize and soybean production levels are partly ascribed to the steady increase in crop yield since the 1960s driven by improvements in genetics, agronomy‐including the implementation of soil conservation measures, and favorable growing conditions (Grassini et al., 2013; Long et al., 2015). However, increase in crop yield over time is a nonlinear process (Ciais et al., 2005) and is subject to stochastic factors such as pest and disease outbreaks and weather (Carvajal‐Yepes et al., 2019; Schlenker & Roberts, 2009). Among these factors, drought has been the major cause of loss in the rainfed “Corn‐Belt” over the last few decades, and water is projected to become increasingly limiting to further potential yield increases (Ort & Long, 2014).

**FIGURE 1:**
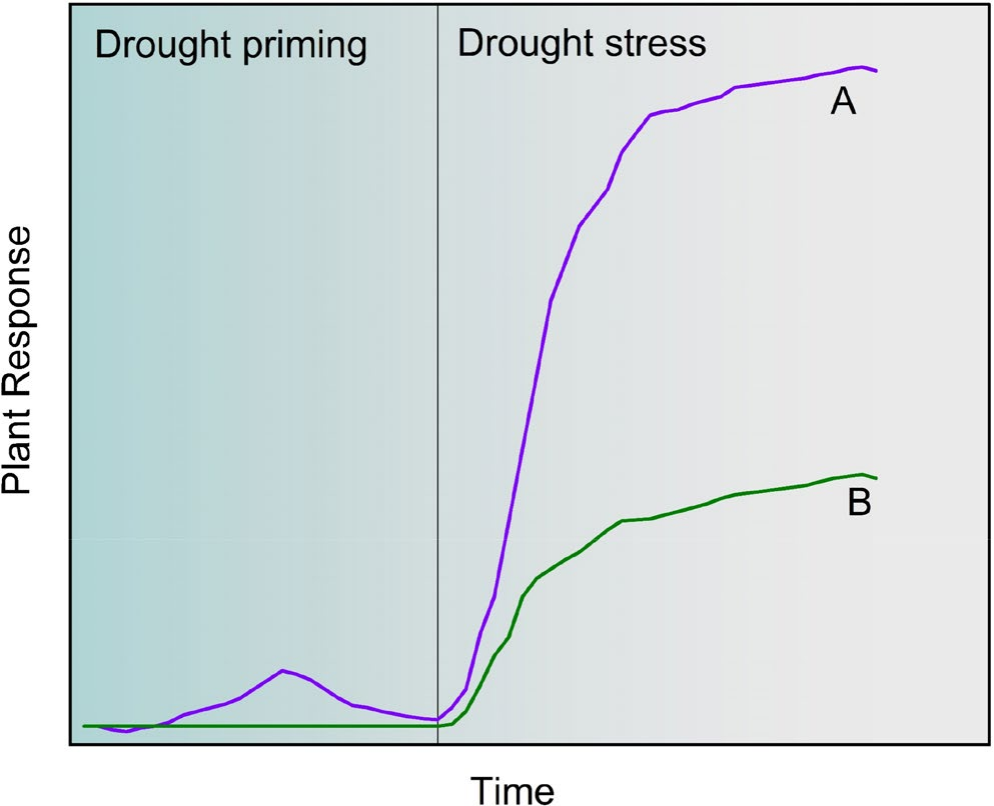
Conceptual illustration of positive response in plants to drought priming and stress. During the drought priming, a plant reacts with altered levels of various metabolites, enzymes, hormones, and other molecules, enabling faster and stronger response/adaption (a) in a drought stress than a plant without priming (b) in a way that can partly mitigate yield losses than nonprimed crops. Altered levels of metabolites, enzymes, hormones, and other molecules may also induce changes in plant traits such as root length, leaf area index, and specific leaf nitrogen as specific phenotypes for drought priming

Despite a trend toward a wetter Midwest, how precipitation is distributed over time is changing with wetter springs and drier summers and early fall (Andresen et al., 2012; S. Dai et al., 2016; Neri et al., 2020). This altered precipitation pattern leads to drier conditions at the peak of leaf area and when crop water demand is greatest. Climate projections show that this trend of wetter springs and drier mid‐ to late‐season conditions in the Midwest will intensify with climate change (USGCRP, 2018), and thus yield losses to drought will likely increase. Recent evidence suggests that stressful growth environments during early vegetative growth stages has little impact on crop yields; however, stress during reproductive development drives reductions in yields (Siebers et al., 2015, 2017). Studies from greenhouse experiments show that a “priming effect” can occur whereby an early‐season drought can minimize impacts on crop growth and yield of a late‐season drought relative to a late‐season drought without an early‐season drought (Balmer et al., 2015; Martinez‐Medina et al., 2016; Wang et al., 2017). Drought primed crops that experience a second drought potentially show a loss of productivity but are generally more resilient in growth and productivity relative to nonprimed crops that experience a drought later in the growing season (Figure 1). Drought primed crops therefore possess the capacity to partly mitigate yield losses relative to nondrought primed crops. Drought priming effects on crops, however, have been tested predominately on plants grown in artificial growth environments at selective crop stages (Mendanha et al., 2020; Wang et al., 2018). Thus, it remains a question as to whether priming occurs under field conditions where greater rooting volumes and other weather variables have significant impacts on crop yield (Chenu et al., 2013; Zhao et al., 2017). If drought priming occurs under field conditions, the current trend toward wetter early growing seasons and drier late growing seasons, minimizing the potential for drought priming, may contribute to the observed increased sensitivity of crop growth to drought in the U.S. Midwest (Lobell et al., 2014). As such, an improved understanding of the priming effect on crops over large spatial extent is necessary, which may help lead to development of more resilient crop cultivars (Balmer et al., 2015; Wang et al., 2018).

Warm‐season temperatures in the Midwest are increasing and are projected to continue rising (USGCRP, 2018). Such a temperature rise, coupled with precipitation change, can cause more surface moisture to be lost through evaporation (Wuebbles et al., 2017). Since increased temperature can also induce high vapor pressure deficit (VPD) that can aggregate drought stress on crops and lead to yield losses in the Midwest (Lobell et al., 2014; Zhao et al., 2017), drought priming effect needs to be differentiated from the potential for a temperature priming effect. This differentiation would further help understand the impacts of temperature and drought on crop production.

Long‐term time series data sets of crop yields coupled with environmental variables have revealed insights into drivers of variability in crop yields (Lobell et al., 2011; McGrath et al., 2015). Reported drought conditions in the United States, for example, by the United States Drought Monitor (USDM), provides opportunity to use historic data to quantify the potential for drought priming to impact crop yield. In this study, satellite images, high‐resolution daily weather observations, county‐level crop yield data, and process‐based crop modeling were used to discern the impacts of drought, with and without a priming response, on crop yield losses. Specifically, satellite time series images were acquired between 2000 and 2018 to derive four key phenological stages for both maize and soybean using a hybrid approach involving a pre‐defined geometric shape fitting (known as shape model fitting, SMF) (Sakamoto et al., 2010) and a threshold method (Zhu et al., 2018). These phenological stages provide the basis to quantify the drought to wet conditions using either the Palmer Drought Severity Index (PDSI) or standardized precipitation anomaly (SPA). Two approaches were used to identify whether the drought priming effect impacted yields, one that relies on a panel regression analysis of actual yield variability by factoring in variables including solar radiation, temperature, precipitation, VPD, and crop phenological stages, and the other that relies on the Agricultural Production System sImulator (APSIM) model (Holzworth et al., 2014) with inputs of selected high‐resolution weather data. The same data were also utilized to identify the temperature priming effect following the approaches used to discern the drought priming effect. The study region consists of three Midwestern states, Illinois, Indiana, and Iowa, in which maize and soybean production are mainly rainfed.

## 2 MATERIALS AND METHODS

### 2.1 Identification of crop phenological stages

**FIGURE 2:**
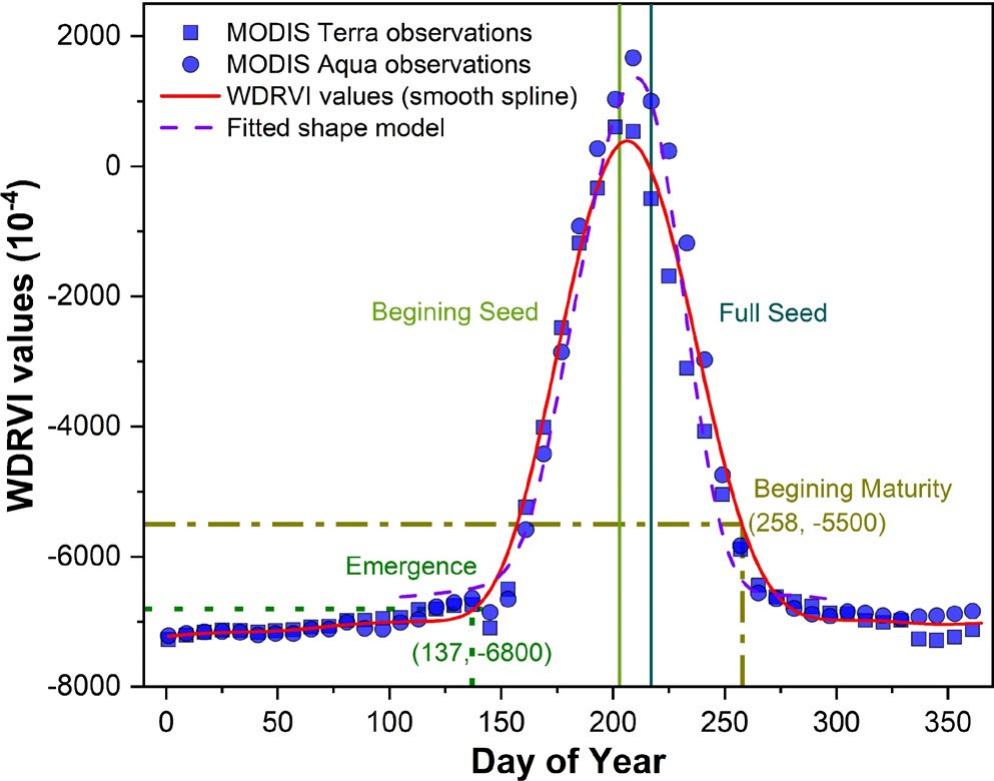
Illustration for the identification of four critical phenological stages using the shape model fitting and threshold method. Data were taken from a soybean pixel. The four stages included emergence (dot green line), beginning seed (light green solid line), full seed (blue solid line), and beginning maturity (dark yellow dash dot line)

The MODIS Version 6 time series reflectance data from Terra and Aqua satellite platforms, that is, MOD09Q1 (2000–2018) and MYD09Q1 (2002–2018), were used to identify crop (soybean and maize) phenological stages. The product provides 8‐day maximum composite surface reflectance at 250 m for MODIS bands 1 (red band) and 2 (near infrared band) that have been corrected for atmospheric conditions such as aerosols and Rayleigh scattering. The MODIS images covering the study area including Illinois, Indiana, and Iowa were mosaicked, re‐projected (WGS84 coordinate system), and then downloaded through the Application for Extracting and Exploring Analysis Ready Samples (AρρEEARS) portal. A hybrid approach involving the shape model fitting (Sakamoto et al., 2010) and the threshold method (Zhu et al., 2018) was used to identify four phenological stages for both soybean and maize (as illustrated in Figure 2). Specifically, the four phenological stages identified for maize include Emergence (VE), Silking stage (R1), Dent stage (R5), and Maturity (R6), and for soybean include Emergence (VE), Beginning seed (R5), Full seed (R6), and Beginning maturity (R7). The shape model fitting was used to identify R1 and R5 for maize and R5 and R6 for soybean, while the threshold method was applied to characterize VE and R6 for maize and VE and R7 for soybean. Although the shape model has the capacity to characterize all four critical phenological stages for maize and soybean, it has been reported that the linear scaling of the shape model cannot help accurately discern patterns and trends in emergence and maturity dates (Zeng et al., 2016; Zhu et al., 2018). Thus, a hybrid approach was used in this study.

Both the shape model fitting and the threshold method were implemented with the scaled Wide Dynamic Range Vegetation Index (WDRVI) (Gitelson, 2004) computed in equation 1:

(1) $$\text{WDRVI}=\left(\alpha *{\text{Ref}}_{b2}‐{\text{Ref}}_{b1}\right)/\left(\alpha *{\text{Ref}}_{b2}+{\text{Ref}}_{b1}\right)$$

where *Ref_b1_* and *Ref_b2_* refer to the MODIS surface reflectance values for bands 1 and 2, and *α* is a weighting coefficient set as 0.1. The calculation of the WDRVI was on a per‐pixel basis and only reflectance values with the highest quality flag were retained for WDRVI calculation. Before the shape model fitting and the threshold method application, the WDRVI curve for each year was smoothed using a spline function to remove any noise from and fill the data gap in the original time series. This smoothing procedure also interpolated the original 8‐day time series into daily smoothed observations.

The reference shape models for maize and soybean were defined by averaging smoothed WDRVI over multiple years (2001–2010) that were acquired over two irrigated field sites at Mead, Nebraska operated by the University of Nebraska Agricultural Research and Development Center (Sakamoto et al., 2010). To identify crop phenological stages, these predefined shape models were scaled and fitted to interpolated daily time series WDRVI data using equation 2:

(2) $$f\left(x\right)=yscale\times h\left(xscale\times \left(x+tshift\right)\right)$$

where *h(x)* is the predefined shape model for maize or soybean, *x* refers to the date, and *xscale*, *tshift*, and *yscale* are variables that need to be optimally determined so as to minimize the difference between *f(x)* and the satellite derived WDRVI curves. Here, the root mean square error (RMSE) between *f(x)* and the satellite‐derived WDRVI curves was used as the loss function which was iteratively minimized with the Levenberg‐Marquardt algorithm (Moré, 1978). The reference dates (Table 1) of four phenological stages for maize were set at 150, 200, 240, and 265, and for soybean at 170, 225, 240, and 270, which were empirically determined based on in situ observations of phenological stages.

**TABLE 1:** Phenological stages identified for maize and soybean using the shape model fitting

Stage	Maize				Soybean			
	Vegetative	Silking	Dent	Maturity	Vegetative	Beginning seed	Full seed	Beginning maturity
	V1	R1	R5	R6	V1	R5	R6	R7
Reference date	150	200	240	265	170	225	240	270

Note

The reference dates were used to define shape models that were geometrically scaled and fitted to time series WDRVI data on a per‐pixel basis.

The threshold values to detect emergence and maturity dates for maize were set at −0.68 and −0.68, and to detect emergence and beginning maturity for soybean were set at −0.68 and −0.55. These threshold values were determined based on trial‐and‐error comparisons between identified dates and United States Department of Agriculture (USDA)/National Agricultural Statistics Service (NASS) reported emergence and maturity dates for all three states. As USDA/NASS weekly Crop Progress Report (CPR) only recorded critical phenological stages (such as emergence and silking) based on area ratios, a sigmoid function was employed to interpolate the area ratio (Figure S1). In situ observations of phenological stages were then set at the date when the interpolated area ratio reached 50% at the state level (Tollenaar et al., 2017). The phenological dates determined from the USDA/NASS CPR were also used to evaluate the accuracy of the shape model fitting and the threshold method to identify the four critical phenological stages.

Another data set used in identifying phenological stages was the NASS cropland data layer (NASS‐CDL) that provided target maize and soybean pixels for implementing the shape model fitting and the threshold method over the three states. The spatial resolution of the NASS‐CDL was generally 30 m but 56 m for data from 2006 to 2009 as different satellite data sets were used to generate the NASS‐CDL. Further details and metadata regarding the CDL dataset can be found in USDA/NASS website (https://www.nass.usda.gov/Research_and_Science/Cropland/metadata/meta.php). The CDL data were aggregated to the same spatial resolution of MODIS images (i.e., 250 m). The shape model fitting and the threshold method were applied only to MODIS pixels with at least 80% maize or soybean fractions (Sakamoto et al., 2010; Zhu et al., 2018). This fraction threshold was to reduce the impact of mixed pixels that may contain signals of nonmaize/soybean information on the identification of crop phenological stages. Finally, the crop phenological stages identified from satellites were aggregated to the county level using the average operation for subsequent analysis. Here we define the durations between neighboring phenological stages as D1, D2, and D3 for maize, that is, D1 for the duration between Emergence and Silking, D2 for the duration between Silking and Dent, and D3 for the duration between Dent and Maturity. For soybean, D1 refers to the duration between Emergence and Beginning Seed, D2 refers to the duration between Beginning Seed and Full Seed, and D3 refers to the duration between Full Seed and Beginning Maturity.

### 2.2 MODIS LAI data

The LAI data used in the study were extracted from the 8‐day 500 m LAI product (i.e., MOD15A2H, available at https://ladsweb.modaps.eosdis.nasa.gov/missions‐and‐measurements/products/MOD15A2H/). This LAI product (2000–present) has been assessed over a widely distributed set of locations and time points and proved to exhibit a high accuracy compared with ground‐truth data. In this study, LAI data covering the three states were mosaicked, re‐projected, and resampled to match the WDRVI data. These LAI data were mainly used to reveal whether differences in LAI exist among different crop groups (nonpriming, priming, and control groups as defined in the section *Drought priming on crops*). To this end, LAI data were aggregated by county and phenological durations (D1‐D3) and the statistical characteristics of LAI data such as mean, median, and standard deviations were reported for comparisons.

### 2.3 Daily weather data

Daily weather data including daily maximum temperature, minimum temperature, accumulative precipitation, downward surface shortwave radiation, mean VPD, and 10‐day PDSI from 2000 to 2018 were downloaded from gridMET (available through the USGS Geo Data portal, https://cida.usgs.gov/gdp/client/#!catalog/gdp/dataset/54dd5df2e4b08de9379b38d8). gridMET provides daily surface meteorological data for the Continental United States at 4 km (Abatzoglou, 2013). It blends the high‐resolution spatial data from PRISM (Daly et al., 2008) with the National Land Data Assimilation System Phase 2 Reanalysis data, resulting in a spatially and temporally continuous product. The product has been extensively validated using weather station networks including RAWS, AgriMet, AgWeatherNet, and USHCN‐2 and has proved to be suitable for landscape ecological modeling (Abatzoglou, 2013).

To be consistent with the data analysis at the county level, the daily weather data were aggregated from 4 km to the county level at which mean values of these meteorological variables were used. These variables were further aggregated to mean values within the three periods, D1, D2, and D3.

### 2.4 Defining drought conditions

We used PDSI and SPA to individually define drought for each of the three periods (D1‐D3) bounded by the four critical phenological stages. PDSI was calculated based on precipitation and temperature data while accounting for changes in soil water content (Alley, 1984; Dai et al., 2004). It is a standardized index typically ranging from −4 (dry) to +4 (wet) albeit that more extreme values are possible. Specifically, PDSI was divided into several groups to indicate meteorological conditions from dry to wet: −4.0 or less (Extreme Drought), −3.0 to −3.9 (Severe Drought), −2.0 to −2.9 (Moderate Drought), −1.9 to +1.9 (Near Normal), +2.0 to 2.9 (Unusual Moist Spell), +3.0 to +3.9 (Very Moist Spell), +4.0 and above (Extremely Moist).

The SPA, as calculated in equation (3), characterizes the degree to which accumulative precipitation deviates from its mean state:

(3) $$x=\frac{\left(x_{y,t}‐\bar{x_{t}}\right)}{\delta }$$

where *x_y_*_,_ *_t_* refers to the mean total precipitation at the county level within each of the three durations (*t*) D1, D2, and D3 in a given year *y* and $\bar{x_{t}}$ represents the multi‐year mean total precipitation within the corresponding duration (*t*). This standardized anomaly has been used before, for example, in Li et al. (2019), to quantify the impacts of excessive rainfall and extreme temperature on the crop yield. Based on this standardized anomaly approach, meteorological dry‐to‐wet events were defined in the following order using the *x* value: −2.0 or less (Extreme Dry), −2.0 to −0.5 (Moderate Dry), −0.5 to +0.5 (Near Normal), +0.5 to +2.5 (Moderate Wet), +2.5 and above (Extreme Wet). The *x* values set for extreme drought and rainfall were uneven because the precipitation distribution showed a longer tail toward high precipitation (Figure S2). These uneven values ensured that extreme drought and rainfall were equally identified and represented (Li et al., 2019).

We also followed the anomaly approach to define temperature conditions (hereafter named as STA) from extreme cold to extreme heat (Li et al., 2019). Similarly, based on the standardized anomaly approach, cold‐to‐heat conditions were defined in the following order using the *x* value: −2.0 or less (Extreme Cold), −2.0 to −0.5 (Moderate Cold), −0.5 to +0.5 (Near Normal), +0.5 to +2.5 (Moderate Heat), +2.5 and above (Extreme Heat).

In this study, we did not use VPD to define drought as it is quite difficult to know the category of drought condition, for example, extreme or moderate drought conditions, based on VPD. However, VPD was used in the panel data analysis as it regulated the behavior of crop stomata and may show impacts of atmospheric conditions on crop yield. The separate quantification of drought conditions using PDSI and SPA provided independent estimates of the impacts of drought priming on crop yield losses.

### 2.5 Crop yield anomaly

The crop grain yield data between 2000 and 2018 at county level for the three states were downloaded from the USDA/NASS Quick Stats 2.0 database and yield trend over years for each county is summarized in Figure S3. The unit for the crop yield data is metric ton per hectare (Mg/Ha). To identify drought priming effects, yield variations induced by weather conditions including the minimum temperature (*T_min_*), maximum temperature (*T_max_*), solar radiation (*Srad*), precipitation (*Precp*), and VPD, as well as the general trend in maize and soybean yield (Figure S3) were removed. Thus, crop yield anomaly was defined after weather‐induce yield variation was removed. Here, crop yield anomaly for each county for a given year was computed in three equations.

First, a panel analysis model, as outlined in equation 4, was used to remove yield variations induced by temperature, solar radiation, and VPD:

(4) $${\text{Yield}}_{i,t}=\frac{\alpha_{0}t+\sum_{j=1}^{3}\left(\alpha_{j}T_{max_{i,t}^{j}}+\beta_{j}T_{min_{i,t}^{j}}\right)+\gamma_{j}{\text{Srad}}_{i,t}^{j}+\mu_{j}{\text{VPD}}_{i,t}^{j}+{\text{County}}_{i}}{{\text{Yield}}_{p,i,t}}+\varepsilon_{i,t}$$

where *Yield_i_*_,_ *_t_* refers to the crop yield data (either maize or soybean) for county *i* in a given year *t*, *α_0_t* characterizes the yield trend ascribed to cultivar development and improved agronomic practices, *j* represents the three durations (D1‐D3), *α_j_*, *β_j_*, *γ_j_* and *μ_j_* defines the sensitivity of crop yield to *T_max_*, *T_min_*, *Srad*, and *VPD*, respectively, within each of the three periods, *County_i_* allows for a separate intercept for each county, which accounts for variation in soil type and agronomic practices in different regions, and *ε_i,t_* is the error term. The computation strategy is noted as C1. We define the difference between the crop yield data (*Yield_i_*_,_ *_t_*) and the crop yield data (*Yield_p_*_,_ *_i_*_,_ *_t_*) provided by equation 4 as the crop yield anomaly (*Yield_ano_*, equation 5).

(5) $${\text{Yield}}_{\mathrm{ano}}={\text{Yield}}_{i,t}‐{\text{Yield}}_{p,i,t}$$

Second, the panel model analysis was still used but without meteorological variables and county intercepts as shown in equation 6. This computation strategy is noted as C2. Equation 6 suggested that only the general trend in crop yield over years for each county was removed. Crop yield anomaly was then defined as the difference between the crop yield data and the expected yield trend (noted as *Yield* *_anoTr_*).

(6) $${\text{Yield}}_{i,t}=\underset{{\text{Yield}}_{p,i,t}}{\underbrace{\alpha_{0}t}}+\varepsilon_{i,t}$$

We made a comparison between the magnitudes of the drought priming effect using yield anomalies derived from C1 and C2 to reveal whether variability in climate conditions would apparently obscure the drought priming effect. Note that the APSIM simulations were driven by weather inputs such as temperature, precipitation, solar radiation, and VPD (similar to the C2 strategy). Thus, the comparison between C1 and C2 would help understand whether APSIM simulated yield data can be directly used (without anomaly calculation as shown in equations 5 and 6) for discerning the drought priming effect.

Third, to check if the temperature (or heat stress) priming effect exists, the panel analysis model (as shown in equation 4) was adjusted with slightly different variables. We repeated analysis steps for the drought priming effect with the temperature data.

(7) $${\text{Yield}}_{i,t}=\frac{\alpha_{0}t+\sum_{j=1}^{3}\left(\beta_{j}T_{min_{i,t}^{j}}+\gamma_{j}{\text{Srad}}_{i,t}^{j}+\mu_{j}{\text{VPD}}_{i,t}^{j}+\varphi_{j}{\text{Precp}}^{j}\right)+{\text{County}}_{i}}{{\text{Yield}}_{p,i,t}}+\varepsilon_{i,t}$$

In this case, the difference between crop yield data and the yield data modeled from equation 7 was used as crop yield anomaly and noted as.*Yield* *_anoTem_*

### 2.6 Analysis of drought and temperature priming effects

If the drought priming effect exists, it can be expected that maize/soybean plants experiencing mild to moderate droughts (as defined by PDSI) in both D1 and D3 but not in D2 would have relatively higher yield than those experiencing droughts only in D3. Similar to the drought priming effect, if the temperature priming effect exists, it can be expected that maize/soybean plants experiencing mild to moderate temperature extremes in both D1 and D3 but not in D2 would have relatively higher yield than those experiencing temperature extremes only in D3. Table 2 shows the delineation of different groups for identifying priming effects on crop yields. To explore the drought priming effect, we selected only a county‐year in which D3 had a PDSI value less than −2.0 (experiencing moderate or extreme drought in the later stage of growth) and D2 had a PDSI value larger than −2.0 but less than +2.0 (near normal condition). The crop yield anomaly data were then grouped by D1 PDSI values in two categories (PDSI<−2 and 0<PDSI<2). The two groups were referred to as priming and no priming groups. The comparison between the two groups would help show the priming effect (if it exists). The two‐sample *t*‐test was used to identify if differences in crop yield anomaly or crop yield between the two groups were statistically significant.

**TABLE 2:** Delineation of different groups, that is, drought/temperature priming, drought/temperature nonpriming, and control group based on PDSI/STA/SPA values within each phenological duration (D1‐D3)

Group	D1	D2	D3
Drought priming	PDSI <−2	−2<PDSI<2	PDSI<−2
Drought nonpriming	−2<PDSI<2	−2<PDSI<2	PDSI<−2
Control group (no drought stress)	−2<PDSI<2	−2<PDSI<2	−2<PDSI<2
Temperature priming	−0.5<STA<+2.0	−0.5<STA<0.5	−0.5<STA<+2.0
Temperature nonpriming	−0.5<STA<0.5	−0.5<STA<0.5	−0.5<STA<+2.0
Control group (no high temperature stress)	−0.5<STA<0.5	−0.5<STA<0.5	−0.5<STA<0.5
Drought priming	−2.0<SPA<−0.5	−0.5<SPA<+0.5	−2.0<SPA<−0.5
Drought nonpriming	−0.5<SPA<+0.5	−0.5<SPA<+0.5	−2.0<SPA<−0.5
Control group (no drought stress)	−0.5<SPA<+0.5	−0.5<SPA<+0.5	−0.5<SPA<+0.5

Note

Both PDSI and SPA values were used to delineate drought priming, nonpriming, and control groups.

The same procedure was also performed using SPA while satisfying the following requirements: for a county‐year being selected, D3 had a SPA value less than −0.5 and D2 had a near normal rainfall condition (−0.5 to +0.5). For a selected county‐year, the corresponding yield anomaly was grouped by SPA in D1 in two categories (−0.5<SPA<+0.5 and −2.0<SPA<−0.5) and then checked if differences in crop yield anomaly between groups were statistically significant using the two‐sample *t*‐test. The two groups were referred to as priming and nonpriming groups.

The same procedure was also applied to group yield anomalies using STA while satisfying the following requirements: for a county‐year being selected, D3 had a STA value greater than 0.5 but less than 2.0 and D2 had a near normal temperature condition (−0.5 to +0.5). For selected county‐year, the corresponding yield anomaly was grouped by STA in D1 in two categories (−0.5<STA<+0.5 and 0.5<STA<2.0) and then checked if differences in crop yield anomaly between groups were statistically significant using the two‐sample *t*‐test. The two groups were referred to as priming and nonpriming groups.

As crop yield anomaly resulted from different modeling results (one of the three *Yield_ano_*, *Yield_anoTr_*, *Yield_anoTem_*), analysis of the priming effect was performed for all the yield anomaly data sets (either temperature or drought). We also define a normal group (or control group) within which crops do not experience either drought or temperature stress over the three durations (D1‐D3). The yield or yield anomalies from this group is considered the attainable nondrought or nontemperature stress yield.

### 2.7 APSIM modeling

A process‐based crop model provides an alternative approach to explore the drought and temperature priming effects and whether the model‐based observations are consistent with satellite‐based observations. Here, the APSIM version 7.10 (Holzworth et al., 2014) was used to simulate crop yield. Specifically, we used the APSIM‐Maize and APSIM‐Soybean modules to simulate crop yield for maize and soybean, respectively. The model simulations were forced with the gridMet weather data that were selected for exploring priming effects on maize or soybean using PDSI, SPA, or STA. For maize simulations, a generic maize hybrid “B_110” provided by APSIM version 7.10 was used, while for soybean simulations, a soybean variety “Pioneer 93M42” was used. Only the county‐year that was previously selected for analyzing drought and temperature priming effect (as suggested by the section *drought imprints on crops*) was simulated in the APSIM model. The soil‐associated variables such as soil organic matter fractions were set as a constant for all county‐year simulations. Specifically, the Clarion soil series determined from the Iowa State University Experimental farm available in the APSIM was used. The use of a constant soil profile for all the simulations thus can help remove variations in crop yield induced by soil characteristics. Sowing dates for both maize and soybean were determined using a variable rule with the sowing window set between 1 May and 15 May based on the model predefined cumulative rainfall and soil water. Sowing density and spacing for maize were set as 10 *plants*/*m^2^* and 0.8 *m* while for soybean were set as 20 *plants*/*m^2^* and 0.6 *m*. As the crop cultivar (either maize or soybean) used in simulations was the same over years, the differences in crop yield between groups as defined by D1 PDSI, SPA, or STA would help identify whether the APSIM model captures the priming effect and whether the quantified effect are similar to that from statistical analysis.

## 3 RESULTS

### 3.1 Accuracy for satellite‐derived phenological dates

Phenological dates for both maize and soybean were identified using a hybrid approach integrating the SMF and the threshold method. The validations at the state level showed that MODIS‐derived dates for phenological stages of maize and soybean (Table 3) were in good agreement with those provided by the NASS. Table 3 shows that the RMSE of the four phenological dates among the three states (Illinois, Indiana, and Iowa) ranged from 2.5 (Silking) to 4.6 days (Dent) for maize, and from 1.4 (Beginning Maturity) to 5.5 days (Emergence) for soybean. These findings were consistent with previous studies in estimating phenological dates for maize and soybean (Sakamoto et al., 2010; Zeng et al., 2016; Zhu et al., 2018), providing a basis to understand whether priming effects exist in maize and soybean.

**TABLE 3:** Accuracy assessment of the four phenological dates identified using the shape model fitting and the threshold method

		Illinois	Indiana	Iowa
Maize	Emergence	4.2	4.5	4.4
	Silking	2.5	3.2	3.9
	Dent	3.7	4.6	4.2
	Maturity	4.3	3.5	4.3
Soybean	Emergence	5.5	5.0	4.8
	Beginning seed	4.1	5.0	3.0
	Full seed	NA	NA	NA
	Beginning maturity	1.4	2.2	2.6

Note

Ground‐truth dates for Full Seed are not available (NA). The numbers indicate the root mean square error (RMSE) between MODIS‐derived phenological dates and NASS reported mean dates at the state level.

### 3.2 Drought and temperature priming effects

**FIGURE 3:**
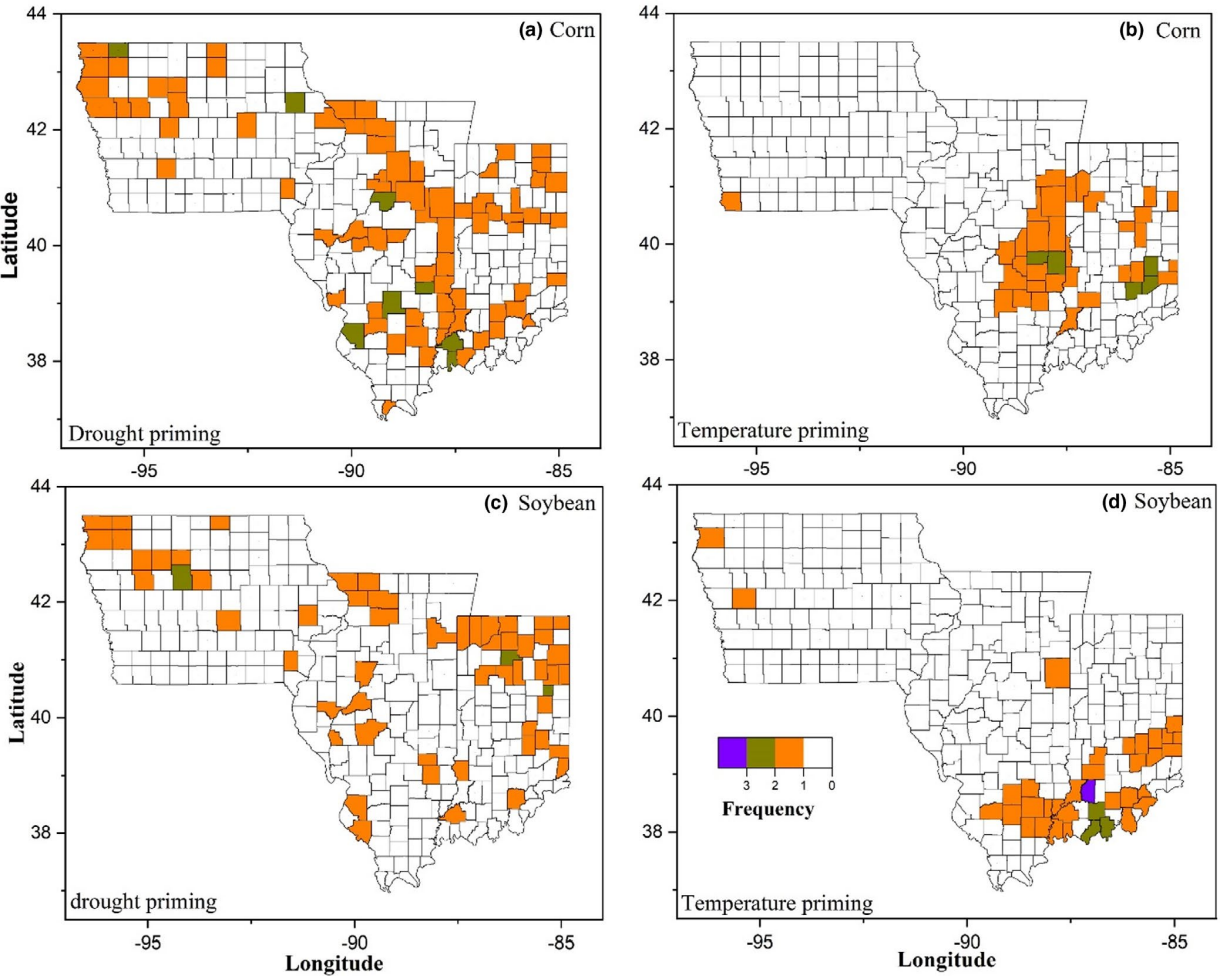
The frequency for a county experiencing a priming event, either drought or temperature, on corn (a, c) and soybean (b, d) from 2000 to 2018. These colored counties are selected using PDSI and STA for exploring whether the priming effect exists

The frequency of a county experiencing the priming event, either drought or temperature, for maize and soybean, is shown in Figure 3. We did not observe a south or north cluster for both temperature and drought priming events although most of the temperature priming events occurred in Illinois and Indiana. Maize and soybean yields from counties experiencing a priming event were compared with those from counties without a priming event to determine if priming effect could mitigate yield loss.

**FIGURE 4:**
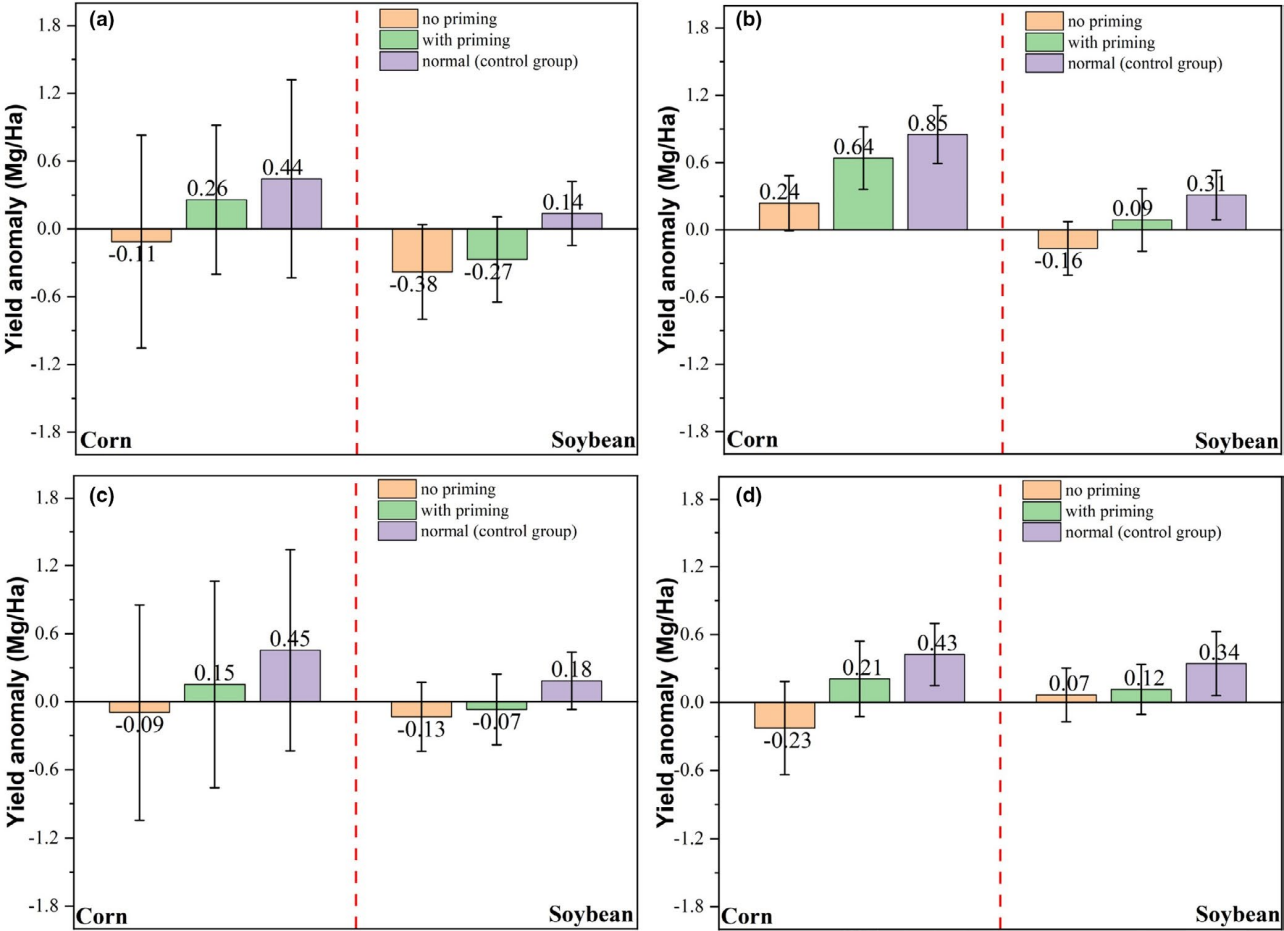
Yield anomalies for maize and soybean without priming, with priming, and in the control group for drought (a and b) and temperature (c and d) conditions. Yield anomalies over time for each county were derived after removing a linear trend from the original yield data (i.e., observed yield minus trend) as shown in (a and c) or after removing the panel analysis modeled yield from the original yield data (i.e., observed yield minus the panel analysis modeled yield) as shown in (b and d). Statistically significant differences between the means of the treatments (no priming, with priming, and control group) for (a) to (d) are observed at a significance level of 0.05 (*p*‐*value* <0.05) using the ANOVA analysis. Numbers close to the bars indicate mean values while the vertical lines with ends represent standard deviations. Drought conditions were determined using the Palmer Drought Severity Index (PDSI) and temperature conditions were determined using the standard temperature anomaly approach. Under normal conditions (also called control group in this study), there is no drought/temperature stress over D1, D2, and D3 and crop yield in this case was considered the attainable nondrought/temperature stress yield

Differences were observed in yield anomalies based on whether crops in a county were exposed to no drought (normal conditions), one drought without priming, or two droughts with a priming event (Figure 4). When the linear trend in yield was removed (Figure 4a), difference in the mean yield anomaly between the drought priming and nonpriming groups was 0.37 Mg/Ha for maize, equivalent to 3.8% of the mean maize yield (9.77 Mg/Ha) across all counties in the three states from 2000 to 2018. The yield anomaly for maize in the control group was 0.44 Mg/Ha, higher than that in the priming group (0.26 Mg/Ha). This suggested that the drought priming effect in maize helped mitigate yield loss by 67.3%. For soybean, the difference in yield anomaly between the drought priming and nonpriming groups was 0.11 Mg/Ha, equivalent to 3.2% of the mean soybean yield (3.41 Mg/Ha) across all counties in the three states from 2000 to 2018. The control group in which no drought stress was observed across the three durations (D1‐D3) showed a yield anomaly of 0.14 Mg/Ha, resulting in a difference of 0.52 Mg/Ha compared with the no priming group and of 0.41 Mg/Ha compared with the priming group. These numbers indicated that the priming effect mitigated the yield loss by 21.2%. When both crop yield trend and yield variations induced by solar radiation and temperature were removed, the drought priming was still observed but with a slightly different magnitude (Figure 4b). For maize, the difference in yield anomaly between the control group and the priming group was 0.21 Mg/Ha and between the control group and the no priming group was 0.61 Mg/Ha. Thus, the priming effect mitigated yield loss by 0.37 Mg/Ha (or 65.6%). For soybean, the priming group reduced the yield loss by 0.25 Mg/Ha (or 53.2%) while the yield loss due to drought was 0.47 Mg/Ha (i.e., the difference between the control group and the no priming group as shown in Figure 4b). This yield loss reduction amounted to 7.3% of the mean soybean yield across all three states from 2000 to 2018. Furthermore, the standard deviation associated with each group decreased from Figure 4a,b. Specifically, standard deviation (unit: Mg/Ha) in the no priming group for maize decreased from 0.94 to 0.24, in the priming group fell from 0.66 to 0.28, and in the normal group declined from 0.88 to 0.26. Soybean showed similar decreases in standard deviation from 0.42 to 0.24, from 0.38 to 0.28, and from 0.28 to 0.22 in the no priming, priming, and normal groups, respectively.

Yield anomalies were also grouped per drought conditions outlined by a SPA approach (Figure S2). We repeated the analysis of the drought priming effect using PDSI with using SPA since previous work showed that PDSI may not always be a good proxy for crop water stress (Woli et al., 2012). Based on Figure S4, the drought priming effect on maize and soybean was still observed with a similar magnitude compared with that identified using PDSI. For example, the difference in maize yield anomaly between the control group and the priming group was 0.21 Mg/Ha and between the control group and the no priming group was 0.62 Mg/Ha when only yield trend was removed (Figure S4a). These numbers suggested that the drought priming effect mitigated the crop yield loss by 0.41 Mg/Ha (or 66.1%), similar to the magnitude derived using the PDSI (67.3%). For soybean, when both yield trend and variations due to temperature and solar radiation were removed, a relatively lower magnitude of the drought priming effect was observed. More specifically, as shown in Figure S4b, the difference in yield anomaly between the priming and no priming groups was 0.21 Mg/Ha, equivalent to 42.0% of the difference in yield anomaly between the normal group and the no priming group. This mitigation of soybean yield loss by 42.0% identified using the SPA approach‐based groups was comparable to the 53.2% loss observed using the PDSI‐based groups.

Following the same approach for precipitation, yield anomalies were further grouped by temperatures to reveal whether temperature priming effects on maize and soybean exist in field conditions. The temperature priming effects for both maize and soybean were identified, evidenced by Figure 4c,d. When only crop yield trend was removed from the original yield data, the difference in yield anomaly between the priming and no priming groups was 0.24 Mg/Ha for maize and was 0.06 Mg/Ha for soybean (Figure 4c). An even higher magnitude for the difference in yield anomaly between the priming and no priming groups was observed for maize (0.44 Mg/Ha) but not for soybean (0.05 Mg/Ha) when both yield trend and variations due to precipitation and VPD were removed using the panel model (Figure 4d). These findings suggested that yield loss mitigated by the temperature priming effect for maize amounted to 2.5% −4.5% of the mean yield across all three states from 2000 to 2018 and for soybean it reached 1.4% −1.8% of the mean soybean yield across all three states from 2000 to 2018. Using yield anomaly in the control group as the reference, the temperature priming effect abated yield loss by 44.4% (Figure 4c)–66.7% (Figure 4d) for maize and reduced yield loss by 18.5% (Figure 4d)–19.4% (Figure 4c) for soybean.

### 3.3 APSIM simulated differences in yield and selected crop traits

Limited in situ and satellite observations are available to reveal differences in plant traits associated with the priming effect at county and state levels. As such, the APSIM model is used first to simulate whether the priming effect exists in field conditions which are different from greenhouse conditions, and then to detect differences in plant traits including LAI, specific leaf nitrogen, and root depth by phenological stages for both maize and soybean. The use of the APSIM model is not to accurately simulate crop yield and plant traits but to provide a further evaluation of what changes in plant traits can be expected. The variation in yield and plant traits, if any, is only driven by the inputs of weather variables as other parameterizations are set as constant variables. The selection of the plant traits including LAI, specific leaf nitrogen, and root depth for analysis in this study is attributable to their importance to explain the fraction of absorbed photosynthetically active radiation, photosynthetic capacity, and accessibility to soil moisture (Adams et al., 2016; Fan et al., 2017; Weiss et al., 2004) that are critical to crop yield.

**FIGURE 5:**
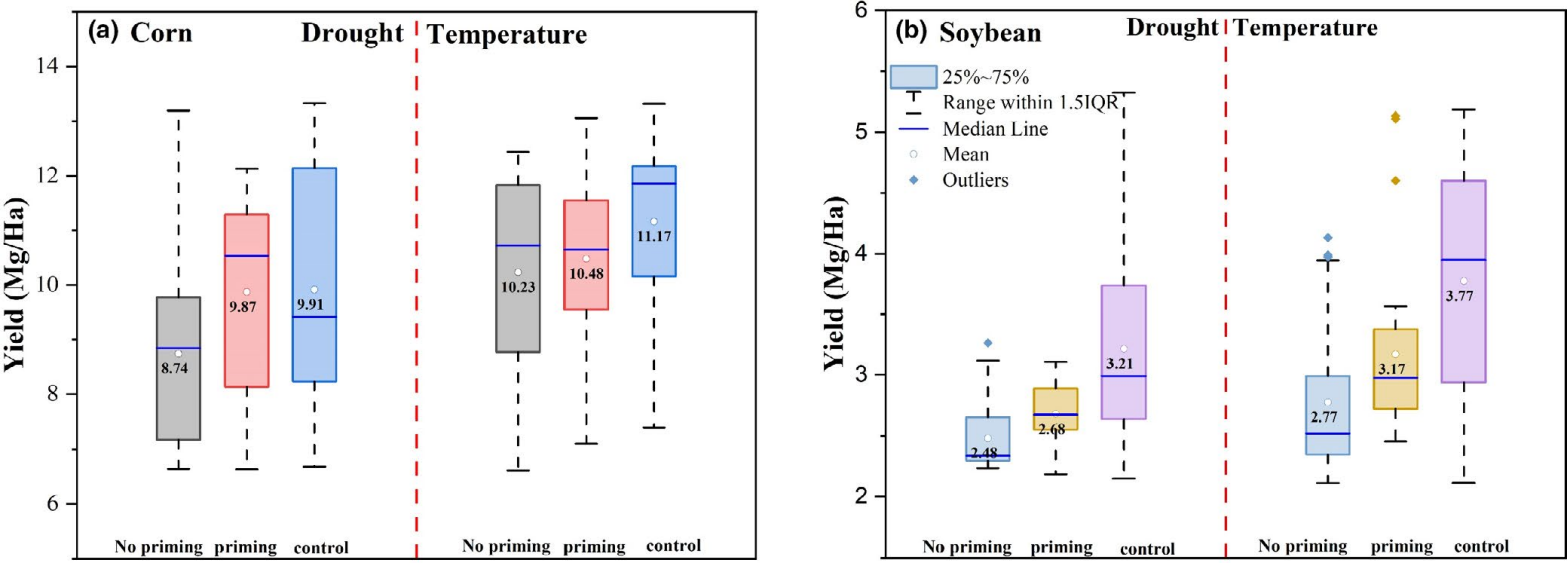
Differences in yield among varying groups (i.e., no priming, with priming, and control) as revealed by the APSIM simulations for maize (a) and soybean (b). Results related to both drought and temperature priming effects are provided (separated by the red dash line). Differences among groups are statistically significant at *p* < 0.05 level

Figure 5 shows simulated yield variation for different groups as revealed by the APSIM model. Both temperature and drought priming effects were explored. For maize, the difference in yield between the drought priming and nonpriming groups was 1.13 Mg/Ha (Figure 5a Left Panel), much larger than that (0.37 Mg/Ha) derived using the panel regression analysis approach while the difference in yield between the drought priming and control groups was relatively smaller (0.04 Mg/Ha, Figure 5a Left Panel, *p*‐*value* >0.05) compared with that (0.18 Mg/Ha) using the panel analysis approach as shown in Figure 4a. With temperature priming, the maize yield was 10.48 Mg/Ha, 0.25 Mg/Ha higher than that in the nonpriming group (10.23 Mg/Ha, Figure 5a right panel). This yield difference between temperature priming and nonpriming groups accounts for 26.6% of the difference in yield between nonpriming and control groups. The temperature priming effect on maize revealed by the APSIM was similar to that (0.24 Mg/Ha) derived using the panel analysis regression‐based approach (Figure 4c,d). For soybean, it was observed that the drought priming effect would help mitigate yield loss by 0.20 Mg/Ha, accounting for 27.4% of the yield loss (i.e., the control group showing a higher yield of 0.73 Mg/Ha compared with the nonpriming group) and roughly 2.0% of the mean yield over all the groups. The temperature priming effect on soybean yield exhibited an even higher magnitude than the drought priming effect as suggested by the APSIM simulations. Specifically, the difference in soybean yield between the temperature control and nonpriming groups was 1.00 Mg/Ha, higher than the difference in yield (0.60 Mg/Ha) between the control and priming groups. These priming effects on soybean were close to those identified using the panel regression analysis‐based approach.

**FIGURE 6:**
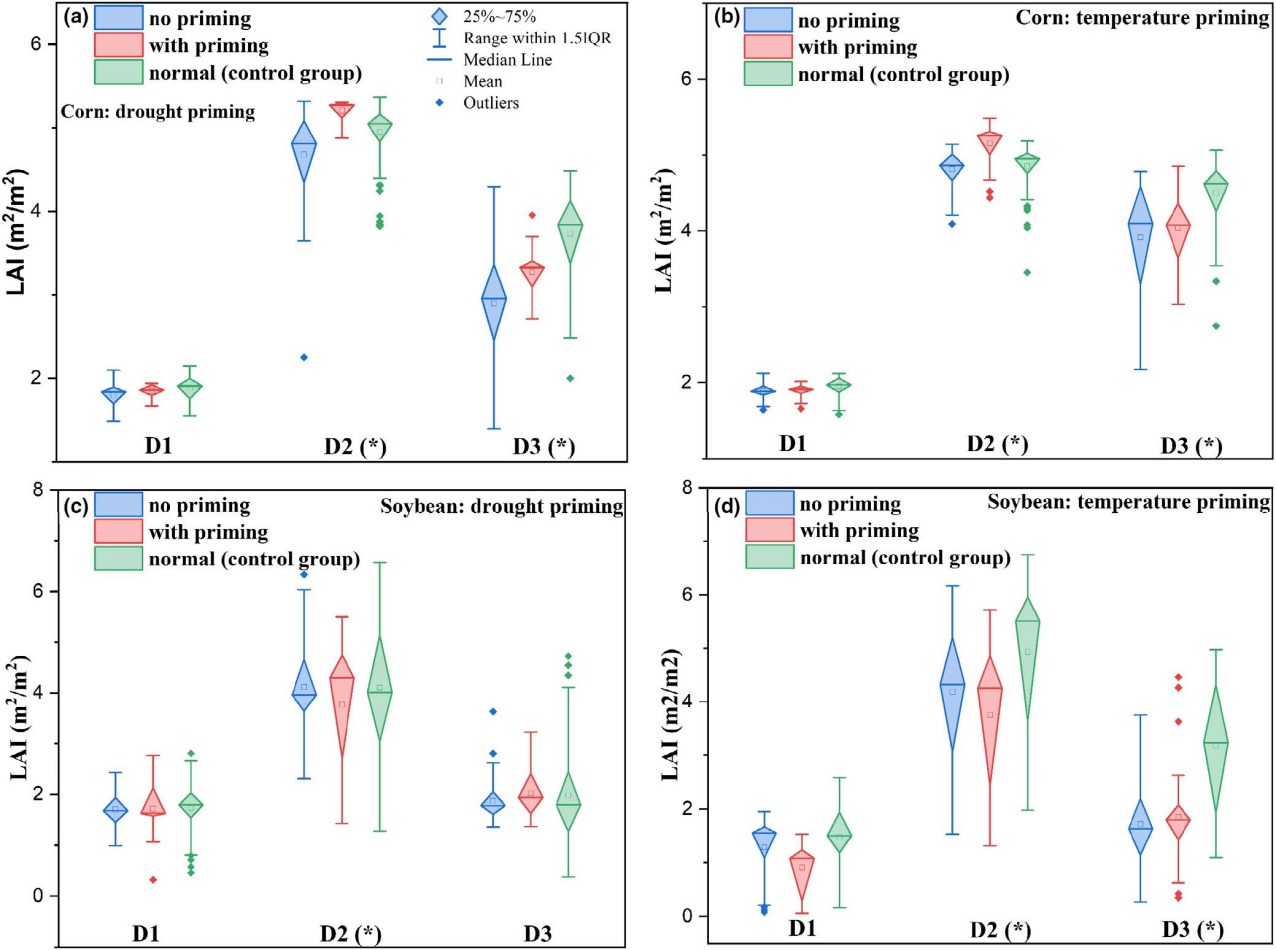
Differences in LAI among groups (i.e., no priming, with priming, and normal) over different crop phenological durations D1, D2, and D3 for both maize (a, b) and soybean (c, d) as revealed by the APSIM model. For maize, D1, D2, and D3 refer to the duration between emergence and silking, silking and dent, and dent and maturity, respectively. For soybean, D1, D2, and D3 refer to the duration between emergence and beginning seed, beginning seed and full seed, full seed and beginning maturity, respectively. *indicates the difference in LAI among groups are statistically significant at *p* < 0.05 level

We also compared the satellite‐derived LAI (MODIS data, Figure S5) and that (Figure 6) provided by the APSIM model (comparisons were made for LAI aggregated over each phenological duration from D1 to D3). The absolute LAI values from the APSIM model differed from the satellite data. However, the difference in LAI values among groups as aggregated by phenological durations (D1‐D3) shared a similar pattern. For example, for maize growth during D2, LAI in the priming group was larger than that in the other two groups (nonpriming and control groups) as revealed both by the APSIM model (Figure 6a,b) and satellite data (Figure S5a,b). For soybean growth during D2, LAI in the priming group (drought and temperature groups) was generally smaller than that in the other two groups as revealed both by the APSIM model (Figure 6c,d) and satellite data (Figure S5c,d). Satellite data generally indicated that LAI did not vary much among groups during D1 and D3 for both soybean and maize (Figure S5) although results from the APSIM model differed slightly from these observations. Further analysis of the APSIM model results showed that only root depth exhibited a statistically significant difference (*p*‐*value*=0.01) between the drought priming group and the other two groups (i.e., nonpriming, priming, and control groups) for both soybean and maize (Figure S6). Specifically, the APSIM model suggested that the drought priming effect induced the increase of root depth by 0.18 m for maize (as compared to the maize nonpriming and control groups) and the increase of root depth by 0.08 m for soybean (as compared with the soybean nonpriming and control groups).

## 4 DISCUSSION AND CONCLUSIONS

Drought is and will continue to be a main factor contributing to yield loss (Lobell et al., 2014). It is imperative to adopt strategies to help crops develop enhanced resistance to drought to ensure global food security for an increasing population while conserving water resources. However, climate change has already aggravated drought intensity and is projected to lead to more frequent extreme weather conditions (e.g., much shorter but intense rainfall events) associated with droughts (Cook et al., 2018). In this study, observational evidence of a drought priming effect was observed on two major Midwestern U.S. crops, maize and soybean. When a late‐season drought was experienced for maize, maize yield losses were 0.37–0.40 Mg/Ha higher (equivalent to $50/Ha–$55/Ha), or roughly 3.8%–4.1% of mean annual yields in the three states (i.e., Illinois, Indiana, Iowa) in the Midwest, compared with those when the crops also experienced an early season drought, supporting the drought priming hypothesis. Similarly, evidence also supported a drought priming effect for soybean to the benefit of 0.11–0.25 Mg/Ha (equivalent to $56/Ha–$128/Ha), or 3.2%–7.3% of mean annual yields in the three states in the Midwest. This finding indicates that maize/soybean experiencing a mild to moderate drought at its early growth stage can have a better capacity to deal with moderate and extreme droughts experienced at its later growth stage. These results are independent of experimental studies in greenhouses where meteorological/agricultural drought conditions occurring in the fields are not easily replicated. The two approaches, the panel regression analysis of actual yield variability and the APSIM simulations, used in this study to discern priming effects, both suggest a similar magnitude of priming effects in temperature and drought on maize and soybean. Thus, results from both approaches corroborate with each other and together point out firmly a promising strategy that can be taken in the future as a supplement to existing methods such as genetic engineering and plant breeding to develop drought‐resistance cultivars (Hu & Xiong, 2014). This supplement to existing methods can be achieved by dissecting changes in plant traits at individual plants and gene levels that underlie the drought priming effect (Balmer et al., 2015).

Since increasing warm season temperatures in the Midwest can lead to surface moisture loss through evaporation (Wuebbles et al., 2017) and then further aggravate drought stress on crops, the temperature priming effects on maize and soybean were also evaluated in this study. Temperature priming effects were revealed on both maize and soybean whereby the impacts of late‐season high temperatures were less severe when crops were exposed to high temperatures early in the season. The temperature priming effect helped mitigate yield loss by 0.24–0.44 Mg/Ha (equivalent to $33/Ha–$60/Ha) for maize and by 0.05–0.06 Mg/Ha (equivalent to $25/Ha–$30/Ha) for soybean, accounting for 2.5%–4.5% of the mean maize yield and 1.4%–1.8% of the mean soybean yield over the Midwestern states. This reduction in yield losses by temperature priming events is comparable to the worldwide average yield loss caused by increased temperatures (Zhao et al., 2017). Thus, the observational evidence presented in this study also corroborates the initial hypothesis that maize/soybean experiencing moderate temperature extremes at its early stage of growth would have a better capacity to deal with extreme and moderate temperatures experienced at its later stage of growth. Moreover, this study moves beyond the current frontier in understanding the impacts of climate change (e.g., temperature and precipitation change) on crop yield by highlighting the importance of understanding the role of increased climate variability on crop resilience.

The APSIM model, despite its general success in reproducing the priming effect on maize and soybean yield, provides slightly different results from those of the panel regression‐based approach as seen from yield and LAI variations. For example, the difference in maize yield between the drought priming and control groups is not statistically significant (*p*‐*value* <0.05) as revealed by the APSIM model simulations (but statistically significant as revealed by the panel regression‐based approach using PDSI or SPA). For soybean, simulation results suggest that the temperature priming effect can help overcome crop yield loss by 0.4 Mg/Ha, much larger than that (0.05–0.06 Mg/Ha) derived from the panel regression analysis‐based approach (Figure 4c,d). These differences between magnitudes of the priming effect derived using the panel regression analysis and the APSIM simulations are potentially explained by APSIM model parameterization using constant variables (e.g., soil conditions were set as constant for all simulations) other than meteorological drivers that determine nonpriming, priming, and control groups. The means by which the APSIM model reproduces the temperature and drought priming effects is likely through stimulation of root growth (Dodd et al., 2008). This effect of the model results in maize and soybean accessing deeper water in the soil profile under stressed conditions, which can have a direct effect on biomass accumulation (Hammer et al., 2009). It is believed that this direct effect on biomass allocation, along with the canopy structure change represented by LAI, may lead to mitigation of yield loss during a priming event. Other process‐based crop models such as those involved in the Agricultural Model Intercomparison and Improvement Project (AgMIP) may also be able to capture this priming effect since most of them have modules to represent responses of plants to weather and soil conditions (Rosenzweig et al., 2014). Additionally, it is found that the APSIM model can replicate the difference in LAI among groups during D2 as compared with satellite‐based LAI. Another possible explanation for the model to reproduce the priming effect, LAI differences, and root depth differences would be the inherent ability of the model to respond to changes in meteorological variables and divert resources to optimize the agricultural productivity (Holzworth et al., 2014). Further studies are needed to improve understanding of how the process‐based models such as APSIM can account for the priming effect or how to explicitly include a mechanism or module to explain the priming effect.

Despite general wetter conditions projected for the Midwestern United States (Neri et al., 2020), such a variation in precipitation patterns may not alleviate drought frequency and intensity for crop growth. Spring precipitation is increasing while precipitation in mid‐ and late‐summer is decreasing, which is likely to lead to fewer opportunities for drought priming making crops more vulnerable to future climate change (Lobell et al., 2014). Because drought priming, as shown in this study, can help lower crop yield loss from late‐season droughts, further understanding of the mechanistic or physiological basis of the drought priming effect is needed. Once these mechanisms are elucidated, the potential may exist to use advanced breeding methods (Hu & Xiong, 2014; Kerchev et al., 2020; Pinto et al., 2010) to identify and incorporate key functional genes into crops, potentially to create “drought primed” cultivars.

Further refinements of this study may be possible by considering the following two aspects. First, although the panel regression analysis reveals drought and temperature priming effects, the magnitude of mitigating crop yield loss exhibits variability due to factors such as soil conditions, irrigation, cultivars, and errors propagated from the satellite‐derived phenological metrics. For example, as the Midwest region is predominately rainfed although rapid irrigation expansion has occurred over the past few years (Xie & Lark, 2021), thus future refinements of this study can be made to exclude a county‐year with large amounts of irrigation over the growing season. As such, the process‐based modeling approach such as APSIM, as a virtual farm platform, would be an alternative approach to understanding the priming effect by setting some variables as constant. However, in this study, despite successful simulations of the temperature and drought priming effects, the APSIM model cannot reproduce all the changes in the plant traits such as LAI over the three phenological durations as compared with satellite observations. This is expected as only one genotype is used in the APSIM model and parameters such as soil conditions are set as constants. Thus, studies are warranted to further understand the priming effects through process‐based crop growth models such as APSIM. Second, the current priming effect is evaluated based on phenological metrics (four key phenological stages for both maize and soybean) derived from 8‐day MODIS composite reflectance images. Thus, it is not possible using current phenological metrics to know precisely when crop priming initiates as conceptually illustrated in Figure 1. It is also difficult to know exactly the amount of drought and temperature stress needed to induce a priming event. Therefore, a precise characterization of when and how the priming effect can be induced may need to combine the greenhouse experimental studies, model simulations, and large‐scale phenotyping of plant traits, biomass, and yield in field conditions, for which further research is required.

## CONFLICT OF INTEREST

The authors declare no conflict of interest.

## Supporting information

Supplementary Material

## References

[fes3332-bib-0001] Abatzoglou, J. T. (2013). Development of gridded surface meteorological data for ecological applications and modelling. International Journal of Climatology, 33(1), 121–131. 10.1002/joc.3413

[fes3332-bib-0002] Adams, M. A. , Turnbull, T. L. , Sprent, J. I. , & Buchmann, N. (2016). Legumes are different: Leaf nitrogen, photosynthesis, and water use efficiency. Proceedings of the National Academy of Sciences, 113(15), 4098. 10.1073/pnas.1523936113 PMC4839396 27035971

[fes3332-bib-0003] Alley, W. M. (1984). The palmer drought severity index: Limitations and assumptions. Journal of Climate and Applied Meteorology, 23(7), 1100–1109.

[fes3332-bib-0004] Andresen, J. , Hilberg, S. , Kunkel, K. , & Center, M. R. C. (2012). Historical climate and climate trends in the Midwestern USA. US National Climate Assessment Midwest Technical Input Report, 1–18. Great Lakes Integrated Sciences and Assessments (GLISA) Center. http://glisa.msu.edu/docs/NCA/MTIT_Historical.pdf

[fes3332-bib-0005] Balmer, A. , Pastor, V. , Gamir, J. , Flors, V. , & Mauch‐Mani, B. (2015). The ‘prime‐ome’: Towards a holistic approach to priming. Trends in Plant Science, 20(7), 443–452. 10.1016/j.tplants.2015.04.002 25921921

[fes3332-bib-0006] Carvajal‐Yepes, M. , Cardwell, K. , Nelson, A. , Garrett, K. A. , Giovani, B. , Saunders, D. G. O. , Kamoun, S. , Legg, J. P. , Verdier, V. , Lessel, J. , Neher, R. A. , Day, R. , Pardey, P. , Gullino, M. L. , Records, A. R. , Bextine, B. , Leach, J. E. , Staiger, S. , & Tohme, J. (2019). A global surveillance system for crop diseases. Science, 364(6447), 1237. 10.1126/science.aaw1572 31249049

[fes3332-bib-0007] Chenu, K. , Deihimfard, R. , & Chapman, S. C. (2013). Large‐scale characterization of drought pattern: A continent‐wide modelling approach applied to the Australian wheatbelt – spatial and temporal trends. New Phytologist, 198(3), 801–820. 10.1111/nph.12192 23425331

[fes3332-bib-0008] Ciais, P. H. , Reichstein, M. , Viovy, N. , Granier, A. , Ogée, J. , Allard, V. , Aubinet, M. , Buchmann, N. , Bernhofer, C. , Carrara, A. , Chevallier, F. , De Noblet, N. , Friend, A. D. , Friedlingstein, P. , Grünwald, T. , Heinesch, B. , Keronen, P. , Knohl, A. , Krinner, G. , … Valentini, R. (2005). Europe‐wide reduction in primary productivity caused by the heat and drought in 2003. Nature, 437(7058), 529–533. 10.1038/nature03972 16177786

[fes3332-bib-0009] Cook, B. I. , Mankin, J. S. , & Anchukaitis, K. J. (2018). Climate change and drought: From past to future. Current Climate Change Reports, 4(2), 164–179. 10.1007/s40641-018-0093-2

[fes3332-bib-0010] Dai, A. , Trenberth, K. E. , & Qian, T. (2004). A global dataset of palmer drought severity index for 1870–2002: Relationship with soil moisture and effects of surface warming. Journal of Hydrometeorology, 5(6), 1117–1130. 10.1175/JHM-386.1

[fes3332-bib-0011] Dai, S. , Shulski, M. D. , Hubbard, K. G. , & Takle, E. S. (2016). A spatiotemporal analysis of Midwest US temperature and precipitation trends during the growing season from 1980 to 2013. International Journal of Climatology, 36(1), 517–525. 10.1002/joc.4354

[fes3332-bib-0012] Daly, C. , Halbleib, M. , Smith, J. I. , Gibson, W. P. , Doggett, M. K. , Taylor, G. H. , Curtis, J. , & Pasteris, P. P. (2008). Physiographically sensitive mapping of climatological temperature and precipitation across the conterminous United States. International Journal of Climatology, 28(15), 2031–2064. 10.1002/joc.1688

[fes3332-bib-0013] Dodd, I. C. , Davies, W. J. , Belimov, A. A. , & Safronova, V. I. (2008). Manipulation of soil: Plant signalling networks to limit water use and sustain plant productivity during deficit irrigation–a review. Acta Horticulture, 792, 233–239. 10.17660/ActaHortic.2008.792.26

[fes3332-bib-0014] Fan, Y. , Miguez‐Macho, G. , Jobbágy, E. G. , Jackson, R. B. , & Otero‐Casal, C. (2017). Hydrologic regulation of plant rooting depth. Proceedings of the National Academy of Sciences, 114(40), 10572. 10.1073/pnas.1712381114 PMC5635924 28923923

[fes3332-bib-0015] FAO . (2019). Food and agriculture organization of the United nations (FAO statistical databases, Rome, 2019). https://www.fao.org/faostat/en/#data

[fes3332-bib-0016] Gitelson, A. A. (2004). Wide dynamic range vegetation index for remote quantification of biophysical characteristics of vegetation. Journal of Plant Physiology, 161(2), 165–173. 10.1078/0176-1617-01176 15022830

[fes3332-bib-0017] Grassini, P. , Eskridge, K. M. , & Cassman, K. G. (2013). Distinguishing between yield advances and yield plateaus in historical crop production trends. Nature Communications, 4(1), 2918. 10.1038/ncomms3918 PMC3905725 24346131

[fes3332-bib-0018] Hammer, G. L. , Dong, Z. , McLean, G. , Doherty, A. L. , Messina, C. , Schussler, J. , Zinselmeier, C. , Paszkiewicz, S. , & Cooper, M. (2009). Can changes in canopy and/or root system architecture explain historical maize yield trends in the U.S. corn belt? Crop Science, 49(1), 299–312. 10.2135/cropsci2008.03.0152

[fes3332-bib-0019] Holzworth, D. P. , Huth, N. I. , deVoil, P. G. , Zurcher, E. J. , Herrmann, N. I. , McLean, G. , Chenu, K. , van Oosterom, E. J. , Snow, V. , Murphy, C. , Moore, A. D. , Brown, H. , Whish, J. P. M. , Verrall, S. , Fainges, J. , Bell, L. W. , Peake, A. S. , Poulton, P. L. , Hochman, Z. , … Keating, B. A. (2014). APSIM – Evolution towards a new generation of agricultural systems simulation. Environmental Modelling & Software, 62, 327–350. 10.1016/j.envsoft.2014.07.009

[fes3332-bib-0020] Hu, H. , & Xiong, L. (2014). Genetic engineering and breeding of drought‐resistant crops. Annual Review of Plant Biology, 65(1), 715–741. 10.1146/annurev-arplant-050213-040000 24313844

[fes3332-bib-0021] Kerchev, P. , van der Meer, T. , Sujeeth, N. , Verlee, A. , Stevens, C. V. , Van Breusegem, F. , & Gechev, T. (2020). Molecular priming as an approach to induce tolerance against abiotic and oxidative stresses in crop plants. Biotechnology Advances, 40, 107503. 10.1016/j.biotechadv.2019.107503 31901371

[fes3332-bib-0022] Li, Y. , Guan, K. , Schnitkey, G. D. , DeLucia, E. , & Peng, B. (2019). Excessive rainfall leads to maize yield loss of a comparable magnitude to extreme drought in the United States. Global Change Biology, 25(7), 2325–2337. 10.1111/gcb.14628 31033107 PMC6850578

[fes3332-bib-0023] Lobell, D. B. , Roberts, M. J. , Schlenker, W. , Braun, N. , Little, B. B. , Rejesus, R. M. , & Hammer, G. L. (2014). Greater sensitivity to drought accompanies maize yield increase in the U.S. Midwest. Science, 344(6183), 516. 10.1126/science.1251423 24786079

[fes3332-bib-0024] Lobell, D. B. , Schlenker, W. , & Costa‐Roberts, J. (2011). Climate trends and global crop production since 1980. Science, 333(6042), 616. 10.1126/science.1204531 21551030

[fes3332-bib-0025] Long, S. P. , Marshall‐Colon, A. , & Zhu, X.‐G. (2015). Meeting the global food demand of the future by engineering crop photosynthesis and yield potential. Cell, 161(1), 56–66. 10.1016/j.cell.2015.03.019 25815985

[fes3332-bib-0026] Martinez‐Medina, A. , Flors, V. , Heil, M. , Mauch‐Mani, B. , Pieterse, C. M. J. , Pozo, M. J. , Ton, J. , van Dam, N. M. , & Conrath, U. (2016). Recognizing plant defense priming. Trends in Plant Science, 21(10), 818–822. 10.1016/j.tplants.2016.07.009 27507609

[fes3332-bib-0027] McGrath, J. M. , Betzelberger, A. M. , Wang, S. , Shook, E. , Zhu, X.‐G. , Long, S. P. , & Ainsworth, E. A. (2015). An analysis of ozone damage to historical maize and soybean yields in the United States. Proceedings of the National Academy of Sciences, 112(46), 14390. 10.1073/pnas.1509777112 PMC4655515 26578785

[fes3332-bib-0028] Mendanha, T. , Rosenqvist, E. , Nordentoft Hyldgaard, B. , Doonan, J. H. , & Ottosen, C.‐O. (2020). Drought priming effects on alleviating the photosynthetic limitations of wheat cultivars (Triticum aestivum L.) with contrasting tolerance to abiotic stresses. Journal of Agronomy and Crop Science, 206, 651–664. 10.1111/jac.12404

[fes3332-bib-0029] Moré, J. J. (1978). The Levenberg‐Marquardt algorithm: Implementation and theory (pp. 105–116). Springer Berlin Heidelberg.

[fes3332-bib-0030] Neri, A. , Villarini, G. , & Napolitano, F. (2020). Statistically‐based projected changes in the frequency of flood events across the U.S. Midwest. Journal of Hydrology, 584, 124314. 10.1016/j.jhydrol.2019.124314

[fes3332-bib-0031] Ort, D. R. , & Long, S. P. (2014). Limits on yields in the corn belt. Science, 344(6183), 484. 10.1126/science.1253884 24786071

[fes3332-bib-0032] Pinto, R. S. , Reynolds, M. P. , Mathews, K. L. , McIntyre, C. L. , Olivares‐Villegas, J.‐J. , & Chapman, S. C. (2010). Heat and drought adaptive QTL in a wheat population designed to minimize confounding agronomic effects. Theoretical and Applied Genetics, 121(6), 1001–1021. 10.1007/s00122-010-1351-4 20523964 PMC2938441

[fes3332-bib-0033] Rosenzweig, C. , Elliott, J. , Deryng, D. , Ruane, A. C. , Müller, C. , Arneth, A. , Boote, K. J. , Folberth, C. , Glotter, M. , Khabarov, N. , Neumann, K. , Piontek, F. , Pugh, T. A. M. , Schmid, E. , Stehfest, E. , Yang, H. , & Jones, J. W. (2014). Assessing agricultural risks of climate change in the 21st century in a global gridded crop model intercomparison. Proceedings of the National Academy of Sciences, 111(9), 3268. 10.1073/pnas.1222463110 PMC3948251 24344314

[fes3332-bib-0034] Sakamoto, T. , Wardlow, B. D. , Gitelson, A. A. , Verma, S. B. , Suyker, A. E. , & Arkebauer, T. J. (2010). A two‐step filtering approach for detecting maize and soybean phenology with time‐series MODIS data. Remote Sensing of Environment, 114(10), 2146–2159. 10.1016/j.rse.2010.04.019

[fes3332-bib-0035] Schlenker, W. , & Roberts, M. J. (2009). Nonlinear temperature effects indicate severe damages to U.S. crop yields under climate change. Proceedings of the National Academy of Sciences, 106(37), 15594. 10.1073/pnas.0906865106 PMC2747166 19717432

[fes3332-bib-0036] Siebers, M. H. , Slattery, R. A. , Yendrek, C. R. , Locke, A. M. , Drag, D. , Ainsworth, E. A. , Bernacchi, C. J. , & Ort, D. R. (2017). Simulated heat waves during maize reproductive stages alter reproductive growth but have no lasting effect when applied during vegetative stages. Agriculture, Ecosystems & Environment, 240, 162–170. 10.1016/j.agee.2016.11.008

[fes3332-bib-0037] Siebers, M. H. , Yendrek, C. R. , Drag, D. , Locke, A. M. , Rios Acosta, L. , Leakey, A. D. B. , & Ort, D. R. (2015). Heat waves imposed during early pod development in soybean (Glycine max) cause significant yield loss despite a rapid recovery from oxidative stress. Global Change Biology, 21(8), 3114–3125. 10.1111/gcb.12935 25845935

[fes3332-bib-0038] Tollenaar, M. , Fridgen, J. , Tyagi, P. , Stackhouse, P. W. Jr , & Kumudini, S. (2017). The contribution of solar brightening to the US maize yield trend. Nature Climate Change, 7(4), 275–278. 10.1038/nclimate3234 PMC6999786 32021656

[fes3332-bib-0039] USDA . (2017). United States department of agriculture (USDA) (2017) quick stats 2.0. U.S. Department of Agriculture, National Agricultural Statistics Service, Washington DC. https://quickstats.nass.usda.gov/

[fes3332-bib-0040] USGCRP . (2018). Impacts, risks, and adaptation in the United States: Fourth national climate assessment, Volume II. "Midwest" pp. 1515. Reidmiller, D.R., C.W. Avery, D.R. Easterling, K.E. Kunkel, K.L.M. Lewis, T.K. Maycock, and B.C. Stewart (eds.). U.S. Global Change Research Program, Washington, DC, USA. 10.7930/NCA4.2018

[fes3332-bib-0041] Wang, X. , Liu, F.‐L. , & Jiang, D. (2017). Priming: A promising strategy for crop production in response to future climate. Journal of Integrative Agriculture, 16(12), 2709–2716. 10.1016/S2095-3119(17)61786-6

[fes3332-bib-0042] Wang, X. , Zhang, X. , Chen, J. , Wang, X. , Cai, J. , Zhou, Q. , Dai, T. , Cao, W. , & Jiang, D. (2018). Parental drought‐priming enhances tolerance to post‐anthesis drought in offspring of wheat. Frontiers in Plant Science, 9, 261. 10.3389/fpls.2018.00261 29545817 PMC5838469

[fes3332-bib-0043] Weiss, M. , Baret, F. , Smith, G. J. , Jonckheere, I. , & Coppin, P. (2004). Review of methods for in situ leaf area index (LAI) determination: Part II. Estimation of LAI, errors and sampling. Agricultural and Forest Meteorology, 121(1), 37–53. 10.1016/j.agrformet.2003.08.001

[fes3332-bib-0044] Woli, P. , Jones, J. W. , Ingram, K. T. , & Fraisse, C. W. (2012). Agricultural reference index for drought (ARID). Agronomy Journal, 104(2), 287–300. 10.2134/agronj2011.0286

[fes3332-bib-0045] Wuebbles, D. J. , Fahey, D. W. , & Hibbard, K. A. (2017). Climate science special report: Fourth national climate assessment, volume I.

[fes3332-bib-0046] Xie, Y. , & Lark, T. J. (2021). Mapping annual irrigation from landsat imagery and environmental variables across the conterminous United States. Remote Sensing of Environment, 260, 112445.

[fes3332-bib-0047] Zeng, L. , Wardlow, B. D. , Wang, R. , Shan, J. , Tadesse, T. , Hayes, M. J. , & Li, D. (2016). A hybrid approach for detecting corn and soybean phenology with time‐series MODIS data. Remote Sensing of Environment, 181, 237–250. 10.1016/j.rse.2016.03.039

[fes3332-bib-0048] Zhao, C. , Liu, B. , Piao, S. , Wang, X. , Lobell, D. B. , Huang, Y. , Huang, M. , Yao, Y. , Bassu, S. , Ciais, P. , Durand, J.‐L. , Elliott, J. , Ewert, F. , Janssens, I. A. , Li, T. , Lin, E. , Liu, Q. , Martre, P. , Müller, C. , … Asseng, S. (2017). Temperature increase reduces global yields of major crops in four independent estimates. Proceedings of the National Academy of Sciences, 114(35), 9326. 10.1073/pnas.1701762114 PMC5584412 28811375

[fes3332-bib-0049] Zhu, P. , Jin, Z. , Zhuang, Q. , Ciais, P. , Bernacchi, C. , Wang, X. , Makowski, D. , & Lobell, D. (2018). The important but weakening maize yield benefit of grain filling prolongation in the US Midwest. Global Change Biology, 24(10), 4718–4730. 10.1111/gcb.14356 29901245

