# Supplementary material for: Drought imprints on crops can reduce yield loss: Nature's insights for food security
Source: Food Energy Secur. 2021 Sep 30;11(1):e332. doi: 10.1002/fes3.332 (PMC9285083; doi:10.1002/fes3.332)
Supplement: Supplementary file 1 — Supplementary Material [file FES3-11-0-s001.docx]

**Appendix** (for additional figures and tables)


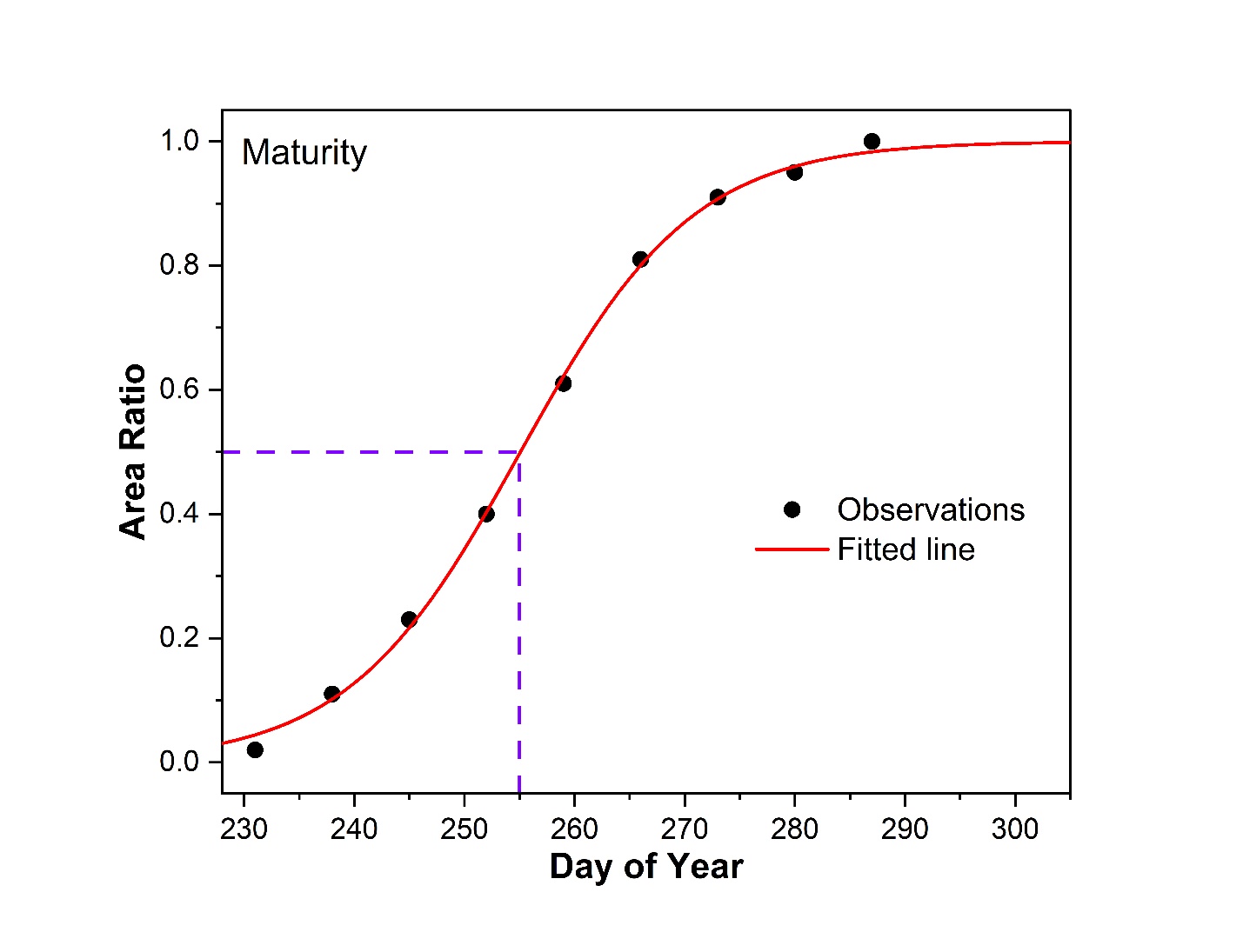


Figure S1. The maturity stage is set at the date when the interpolated area ratio reaches 50% (purple dash line) at the state level. The data presented here are derived from USDA/NASS weekly report that details the area ratio for the maturity stage (black dots). The fitted line is provided by the sigmoid function fitted to the observations. This process was repeated for identifying other phenological stages for both maize and soybean. These phenological dates were used as ground-truth for validating those identified using the shape model fitting and threshold method at the state level.


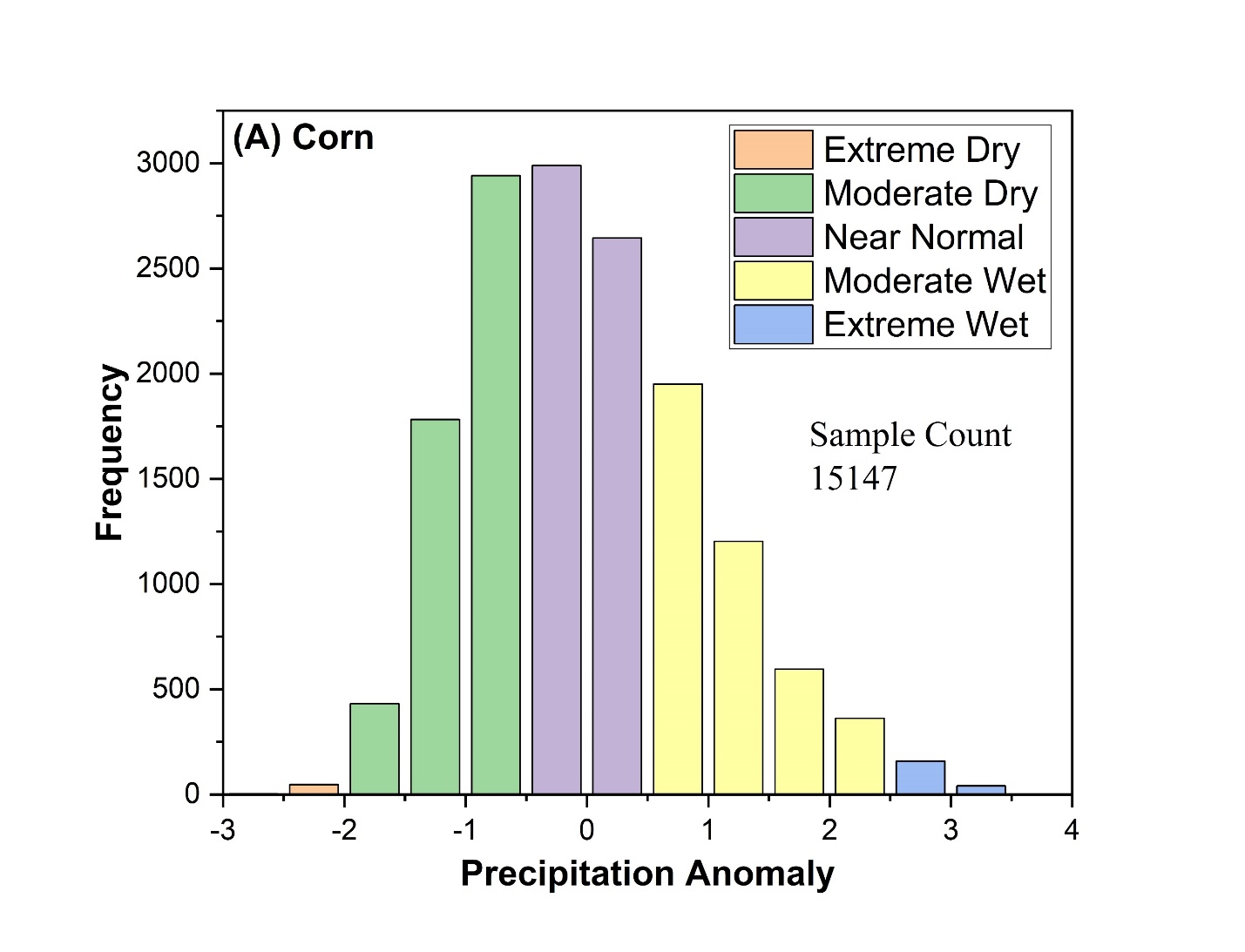


Figure S2. Meteorological dry to wet events defined using the standardized precipitation anomaly. Data used here are accumulative precipitation (2000 - 2018) within each of the three periods bounded by the four phenological stages of maize.


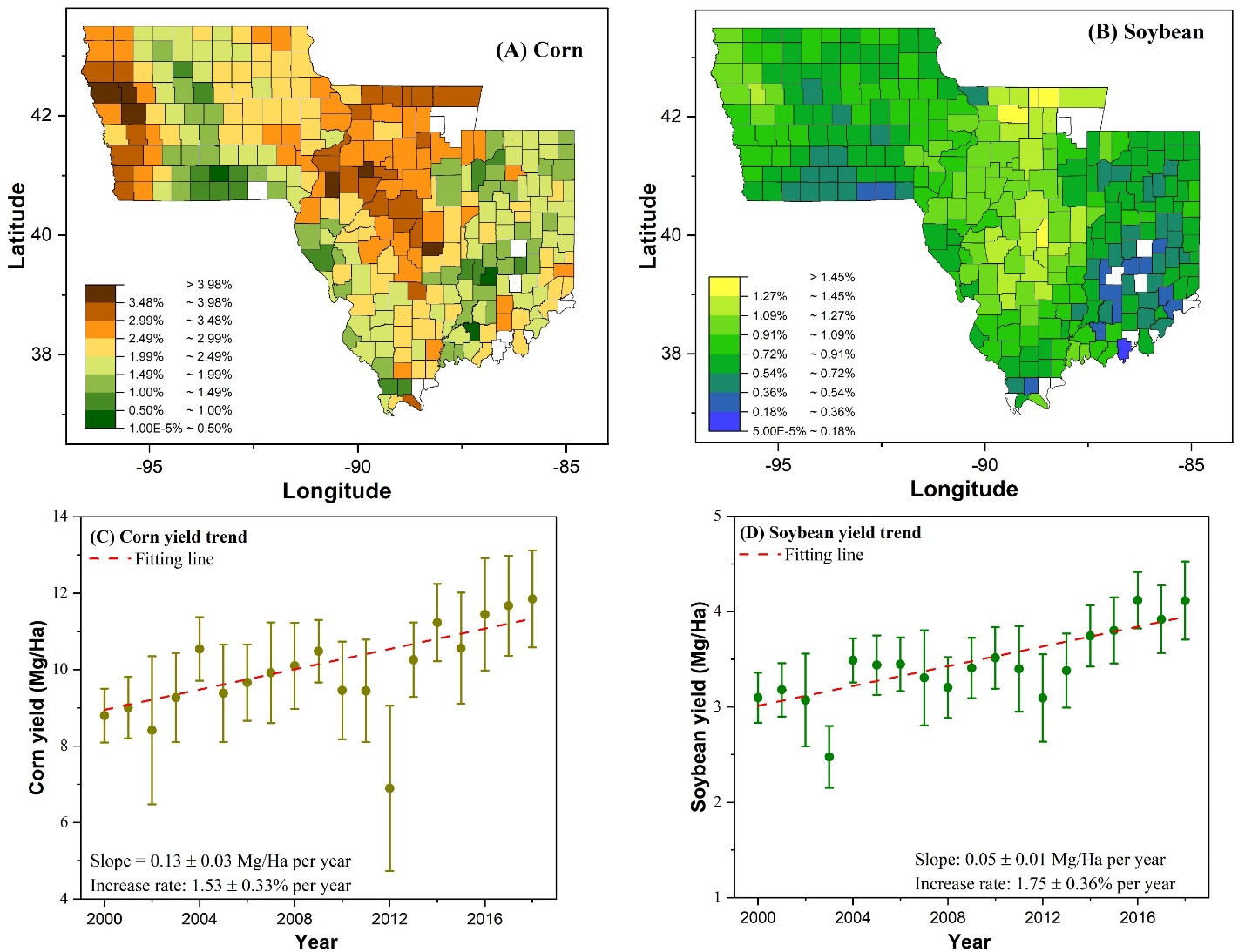


Figure S3. Yield trend, i.e., absolute yield trend and relative yield trend (as shown in percentage), of maize (A) and Soybean (B) for each county. White area in A and B refers to a county with less than 15 years of crop yield data available between 2000 and 2018. C and D provide mean yield trend for all counties for maize and soybean, respectively. The error bars indicate spatial variation of crop yield (minimum to maximum) among all counties.


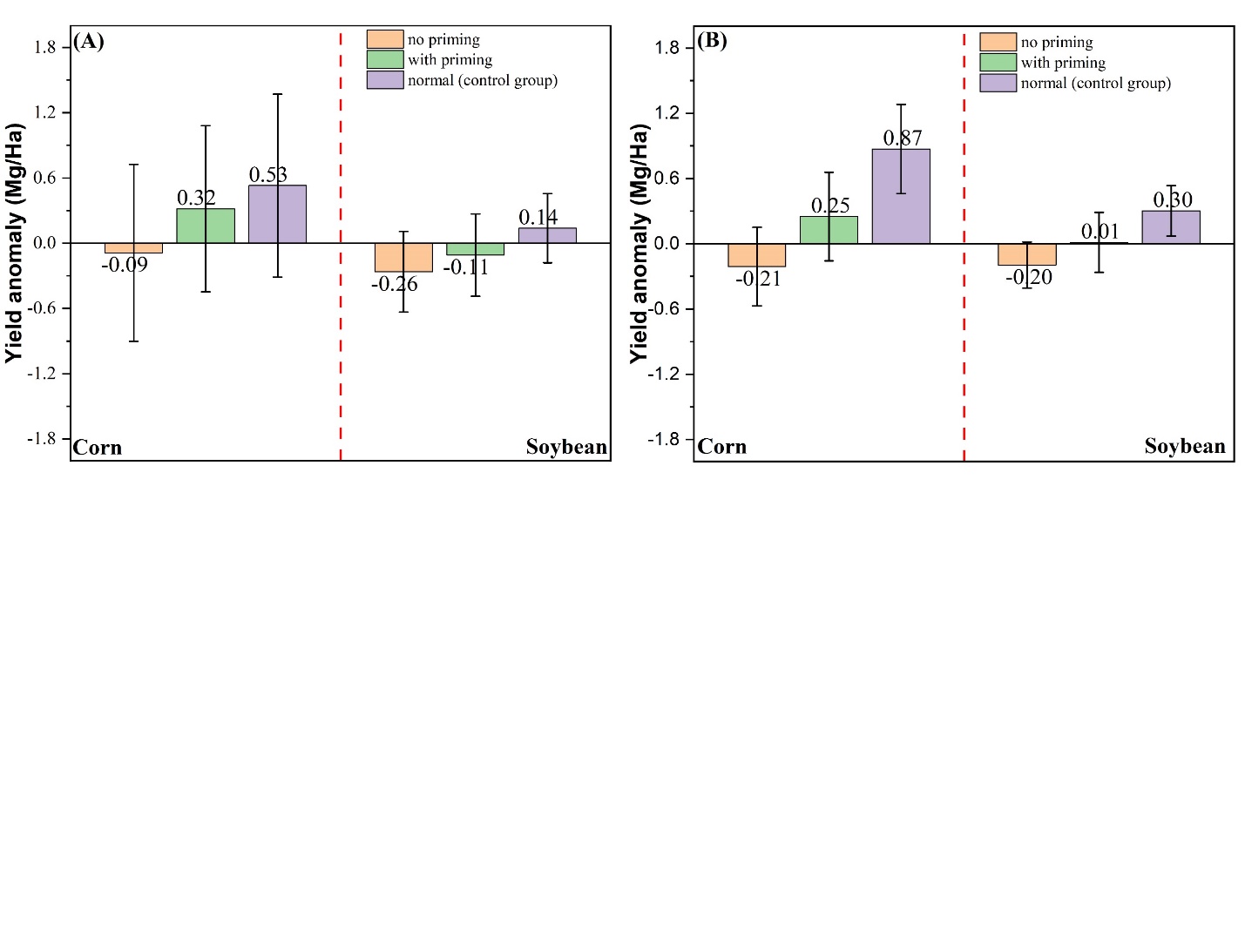


Figure S4. Yield anomalies for crops, i.e., maize and soybean, without priming, with priming, and in the control group (i.e., no drought stress through the growth cycle). Yield anomalies over time for each county were derived after removing a linear trend from the original yield data (i.e., observed yield minus trend) as shown in (A) or after removing the panel analysis modeled yield from the original yield data (i.e., observed yield minus the panel analysis modeled yield) as shown in (B). Statistically significant differences between the means of the treatments (no priming, with priming, and control group) for both (A) and (B) are observed at a significance level of 0.05 (*p-value* < 0.05) using the ANOVA analysis. Numbers close to the bars indicate mean values while the vertical lines with ends represent standard deviations. Drought conditions were determined using the standard precipitation anomaly (SPA).


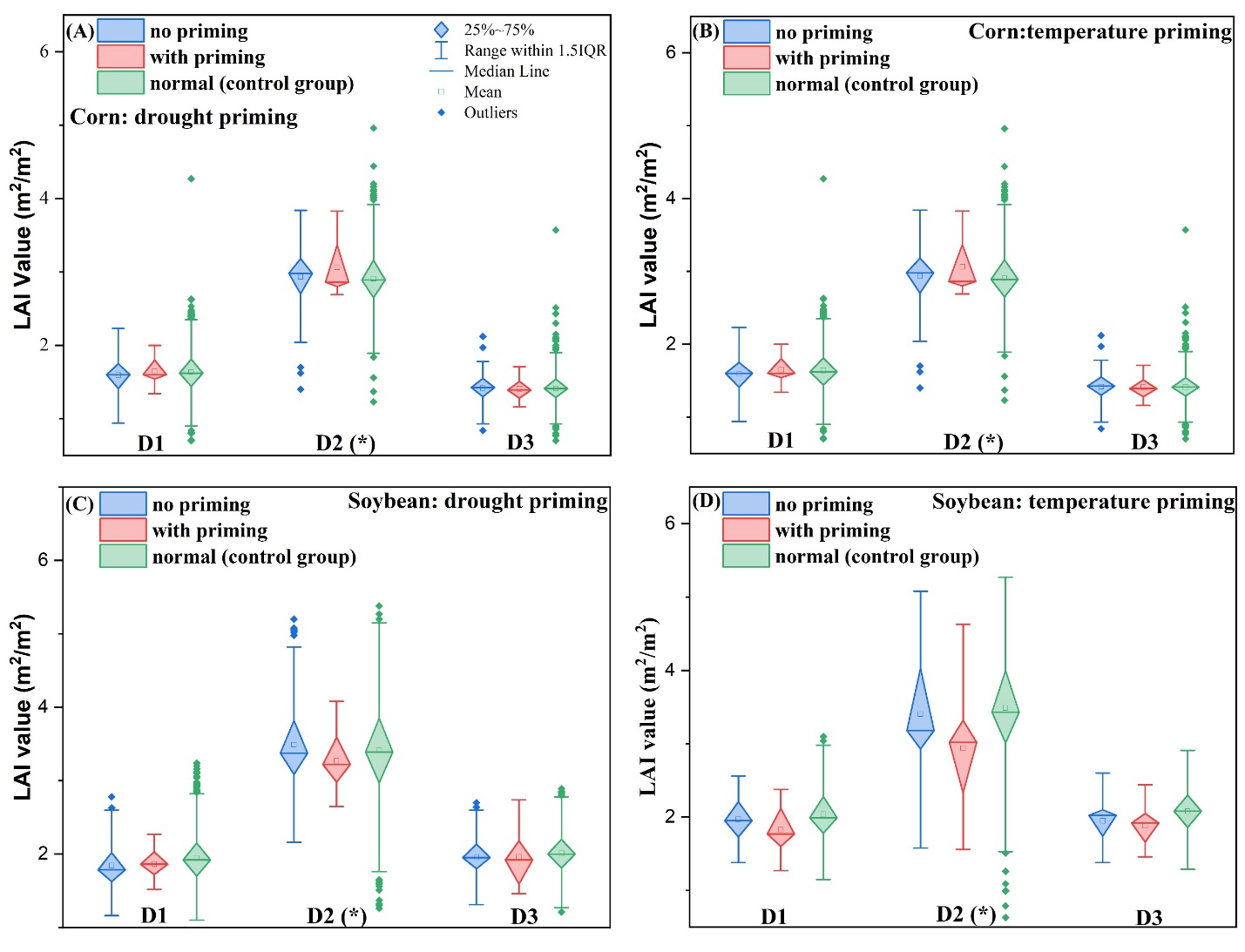


Figure S5. Differences in LAI among groups (i.e., no priming, with priming, and normal) over different crop phenological durations D1, D2, and D3 for both maize and soybean as revealed by the MODIS LAI product. For maize, D1, D2, and D3 refer to the duration between emergence and silking, silking and dent, and dent and maturity, respectively. For soybean, D1, D2, and D3 refer to the duration between emergence and beginning seed, beginning seed and full seed, full seed and beginning maturity, respectively. * indicates the difference in LAI among groups are statistically significant at *p < 0.05* level.


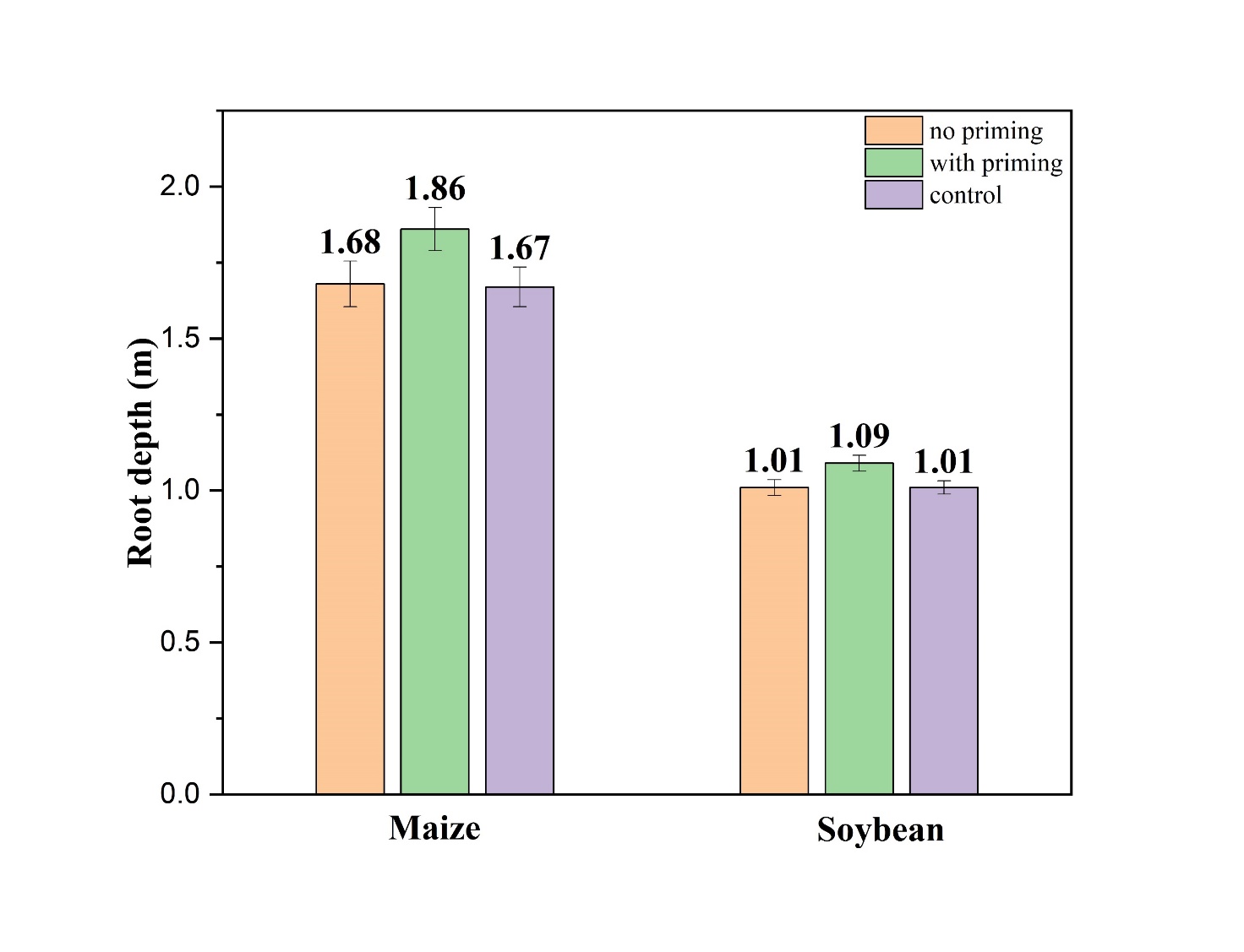


Figure S6. Differences in root depth among groups (no priming, priming, and control groups) as revealed by the APSIM model. The root depth values represent the mean of the root depth of maize or soybean during the phenological duration D2. The differences between the priming group and the other two groups are statistically significant at P < 0.05 level.

Table S1. Phenological stages identified for maize and soybean using the shape model fitting

|  | Maize | | | | Soybean | | | |
| --- | --- | --- | --- | --- | --- | --- | --- | --- |
| Stage | Vegetative | Silking | Dent | Maturity | Vegetative | Beginning Seed | Full Seed | Beginning Maturity |
|  | V1 | R1 | R5 | R6 | V1 | R5 | R6 | R7 |
| Reference Date | 150 | 200 | 240 | 265 | 170 | 225 | 240 | 270 |

Note: the reference dates were used to define shape models that were geometrically scaled and fitted to time series WDRVI data on a per-pixel basis.
